# Gateway to Sustainable Polymers via Catalytic ROCOP of CO_2_/COS Utilizing a Renewable Epoxide Monomer from Furfural Derivatives

**DOI:** 10.1021/acssuschemeng.5c08323

**Published:** 2025-10-08

**Authors:** Sriparna Sarkar, Mani Sengoden, Chia-Min Hsieh, Peiran Wei, Sarnali Sanfui, Donald J. Darensbourg

**Affiliations:** † Department of Chemistry, 14736 Texas A&M University, College Station, Texas 77843, United States; ‡ Soft Matter Facility, Texas A&M University, College Station, Texas 77843, United States

**Keywords:** ROCOP, furan-based monomer, furfural, aliphatic polycarbonates, lignocellulosic biomass

## Abstract

In recent years, there has been an increase in demand for a paradigm shift from fossil fuel-based feedstock to renewable feedstock for polymer synthesis due to the need for expanding the source of these materials as well as enhancing their (bio)­degradability. Lignocellulosic biomass can serve as a promising renewable feedstock that can be converted to platform chemicals. Furfural is one of the crucial platform chemicals that is derived from xylan-rich lignocellulosic biomass. Furfural is an excellent molecular platform that can open a gateway to a diverse range of furan-based epoxide monomers. Herein, we report a furan-based epoxide monomer synthesized by using two furfural derivatives (furoyl chloride and furoic acid). The epoxide has been screened for catalytic ring-opening copolymerization (ROCOP) with carbon dioxide (CO_2_) and carbonyl sulfide (COS) employing binary catalysts comprising Co-salen and Cr-salen complexes, respectively, in conjugation with phosphonium salts. The polycarbonates and poly­(monothiocarbonates) showed thermal stability up to 150 °C with *T*_g_s of 58.6 and 49.2 °C, respectively. The aliphatic polycarbonate exhibited hydrolytic degradability under basic conditions to produce diol. The diol generated by the degradation of polycarbonate was recycled back to epoxide monomer through tosylation reaction, followed by ring-closing of the tosylated product. The mechanical properties of the polymer sample were accessed by performing a lap shear test and nanoindentation testing. The lap shear test revealed that the polycarbonate sample exhibited better adhesive performance than poly­(monothiocarbonates), while nanoindentation testing showed poly­(monothiocarbonates) exhibited higher hardness and elastic modulus than polycarbonates.

## Graphical abstract

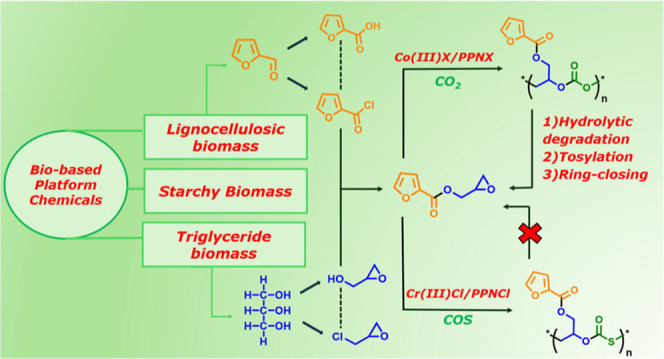

## 1 Introduction

In the 21st century, it is nearly impossible to envision our daily lives without using plastics. The rapid growth in the use of plastics has led to worldwide concern as most of the plastics used are derived from nonbiodegradable polymers, and thus, the end-life of plastics results in white pollution, which ultimately leads to severe environmental damage. Furthermore, most nonbiodegradable polymers are derived from nonrenewable feedstocks, which is eventually detrimental to sustainable development.^1^ The utilization of biodegradable polymers can reduce the environmental burden due to the widespread use of conventional plastics. The aliphatic polycarbonates synthesized by the ring-opening copolymerization (ROCOP) of epoxide with carbon dioxide (CO_2_) have emerged as a promising biodegradable polymer.^2^ In addition, ROCOP is an efficient pathway that supports the emerging carbon capture and utilization (CCU) economy, as CO_2_ emissions can serve as feedstock to produce degradable polymers. Over the years, there have been remarkable discoveries pertaining to the synthesis of CO_2_-based polycarbonates,^3^ and a substantial portion of research efforts have been directed toward the copolymerization of CO_2_ with epoxides derived from petroleum feedstock such as cyclohexene oxide (CHO) and propylene oxide (PO).^4^ The use of epoxides derived partially or completely from biobased sources can play a key role in reducing dependence on petroleum-based sources for synthesizing CO_2_-based aliphatic polycarbonates. The first report on biobased polycarbonates came from Coates and co-workers, where they employed limonene oxide (epoxide derived from orange peel) to synthesize aliphatic polycarbonates using a β-diimino zinc complex as the catalyst.^5^ Subsequently, over the years, different research groups have successfully synthesized polycarbonates with monomers derived from different biobased sources that mainly include sugar, lignin, terpene, essential oils, and vegetable oil.^6–12^ Among various epoxides derived from biobased sources for the synthesis of polycarbonates, the proportion of epoxides derived from nonfood bioderived sources is marginal. It can thus be a promising research direction.^13^ The advantage of epoxides derived from nonfood biomass is that raw material costs are low, and competition with land utilization for ecosystem services is also reduced.^14^ Lignocellulose (obtained from nonedible parts of plants) is an excellent source of nonfood biomass resources on earth.^15^ Furfural is one of the attractive lignocellulose-based molecules,^16^ and thus, epoxides synthesized using furfural derivatives will be advantageous for synthesizing aliphatic polycarbonates from the sustainability viewpoint. Furfural has been recognized as a platform chemical obtained from biomass by the US Department of Energy (DOE).^17^ Furfural is commercially produced from pentosans, mainly xylan, which is present in the hemicellulose of lignocellulosic materials.^18^ The acid-catalyzed hydrolysis of xylan produces xylose, which, on dehydration at higher temperatures, gives furfural. Furan, 2-methylfuran, furfuryl alcohol, tetrahydrofuran (THF), furoyl chloride, and furoic acid are some common furfural derivatives (Figure 1).

**Figure 1:**
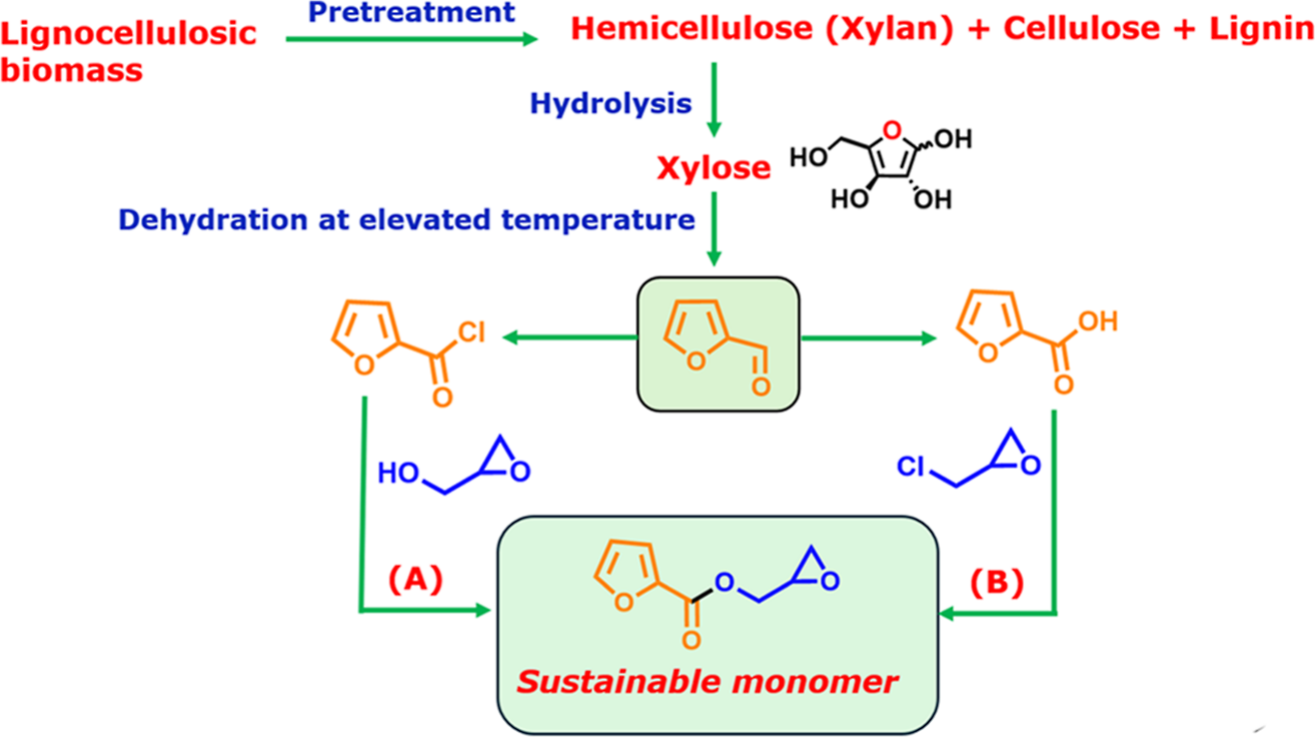
Lignocellulosic biomass as a gateway to sustainable monomer.

In this article, we synthesized an epoxide via two synthetic pathways starting from furfural derivatives furoyl chloride and furoic acid (Figure 1).^19,20^ It is noteworthy to mention that glycidol (pathway A) and epichlorohydrin (pathway B), used in the synthesis of epoxide, can be obtained from renewable feedstock glycerol.^21^ The epoxide has been employed for the synthesis of aliphatic polycarbonates, cyclic carbonate, and poly­(monothiocarbonates) employing Co­(III)- and Cr­(III)-based catalysts, respectively (Figure 2), in conjunction with phosphonium salts as a cocatalyst. The study is remarkable as the epoxide gets added to the limited list of epoxides that produce COS-based copolymers. To date, there have been reports about the catalytic coupling of the epoxide (used in this study) with CO_2_ to produce cyclic carbonates using aluminum- and lanthanum-based catalysts^22,23^ (Figure 2), but no reports are there for CO_2_- and COS-based aliphatic polymers. Furthermore, we investigated the thermal, mechanical, and hydrolytic degradability of polycarbonate and poly­(monothiocarbonate).

**Figure 2:**
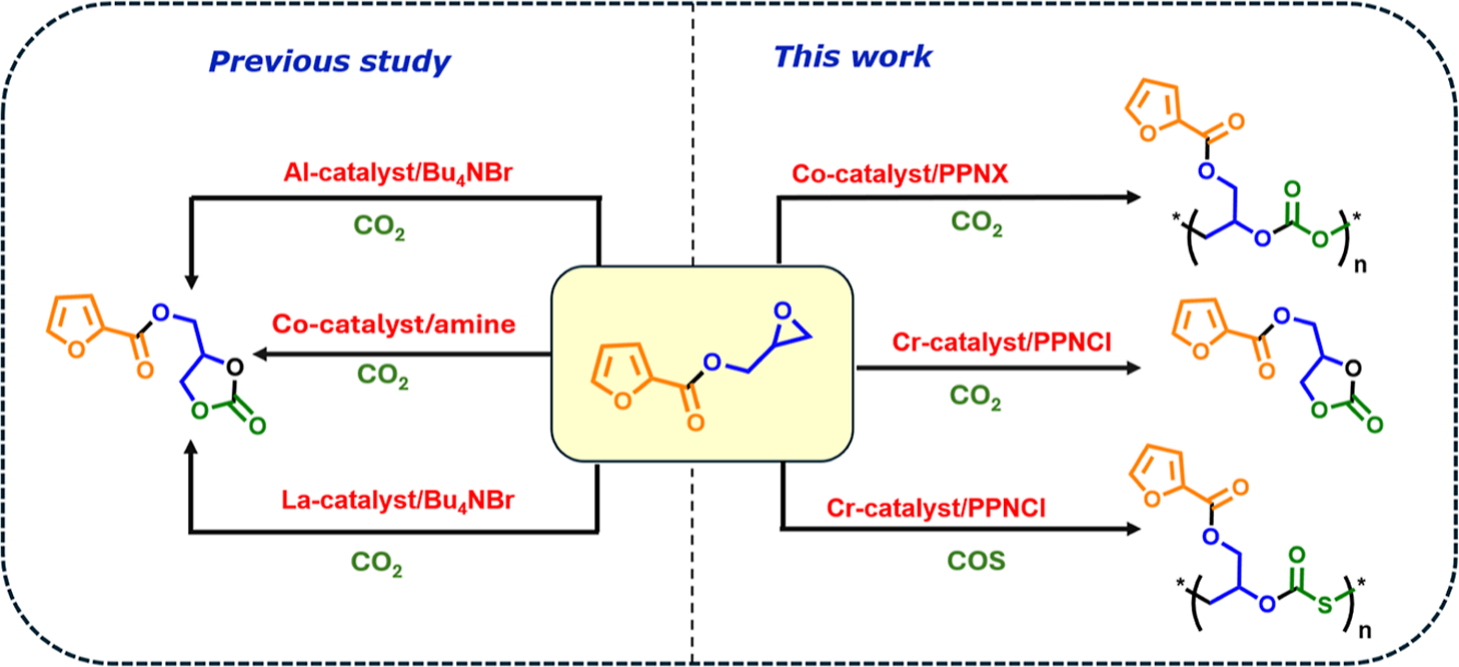
Overview of the previous literature reports and present work.

## 2 Experimental Section

### 2.1 Materials

All the air- and moisture-sensitive reactions were done under an argon or nitrogen atmosphere using standard Schlenk line and glovebox techniques. The glassware used for the reactions was washed thoroughly and oven-dried at 150 ^°^C for 24 h prior to use. The solvents used were purchased commercially and dried in an MBraun Manual Solvent purification system packed with an Alcoa F200 activated alumina desiccant. The starting materials for the synthesis of glycidyl furoate (GFu) were purchased from different commercial sources. 2-Furoic acid and 2-furoyl chloride were purchased from Ambeed, epichlorohydrin was purchased from Thermo Scientific Chemicals, glycidol and triethylamine (NEt_3_) were purchased from Acros, tetrabutylammonium bromide (TBAB) was purchased from Sigma-Aldrich, and sodium hydroxide (NaOH) was purchased from Alfa Aesar. The chemicals needed for the synthesis of ligand -3,5-di-*tert*-butyl 2-hydroxybenzaldehye, ethylene diamine, and 4-methyl-o-phenylenediamine were purchased from Sigma-Aldrich. The chemicals required for the preparation of Co­(III) metal complexes, Co­(OAc)_2_·4H_2_O, (*R*,*R*)-*N*,*N*′-bis­(3,5-di-*tert*-butylsalicylidene)-1,2-cyclohexanediamino-cobalt­(II) was purchased from Strem Chemicals and silver trifluoroacetate was purchased from Sigma-Aldrich. (*R*,*R*)-*N*,*N*′-Bis­(3,5-di-*tert-*butylsalicylidene)-1,2-cyclohexanediamino-chromium­(III) chloride was purchased from Strem Chemicals. Among the three cocatalyst used for the copolymerization reaction, bis­(triphenylphosphine)­iminium chloride (PPNCl) and bis­(triphenylphosphine)­iminium trifluoroacetate (PPNTFA) were purchased from commercial sources and bis­(triphenylphosphine)­iminium azide (PPNN_3_) was prepared in the laboratory according to the literature reported procedure.^24^ Carbon dioxide (CO_2_) gas was purchased from Conroe Welding and >99% carbonyl sulfide from Praxair. The deuterated chloroform (CDCl_3_) for the NMR analysis was purchased from Thermo Scientific Chemicals.

### 2.2 Methods

The NMR spectra were recorded on a 400 MHz Bruker spectrometer with CDCl_3_ as an internal standard reference at 7.26 ppm. Infrared spectra were recorded using a Bruker Tensor 27 FT-IR spectrometer and CaF_2_ sample cell with a 0.02 mm path length. The Gel Permeation Chromatography (GPC) studies to determine the molecular weight of the polymer were performed using a Malvern modular GPC apparatus with ViscoGEL I-series columns (H&L) and THF as an eluent. *M*_w_ and *M*_n_ were calculated using data from Refractive Index (RI), Right Angle Light Scattering (RALS) and Low Angle Light Scattering (LALS) detectors calibrated against polystyrene standards. MALDI-TOF analysis for the polymer samples was done in a Bruker Microflex MALDI-TOF instrument.

#### 2.2.1 Thermal Characterization

Thermogravimetric analysis for the polymers was performed on a Mettler-Toledo TGA/DSC 1 analyzer. The polymeric samples were heated from room temperature to 500 °C at a rate of 10 °C·min^–1^ under a N_2_ flow of 20 mL·min^–1^. DSC measurements were performed on a TA Instruments DSC2500. Temperature and heat flow were calibrated by an indium standard. Ramp 10.00 °C/min to 25.00 °C; isothermal for 1.0 min; Ramp 10.00 °C/min to 130.00 °C; isothermal for 1.0 min; Ramp 10.00 °C/min to 25.00 °C; isothermal for 1.0 min; Ramp 10.00 °C/min to 130.00 °C; isothermal for 1.0 min; Ramp 10.00 °C/min to 25.00 °C (three cycles). The *T*_g_ was taken as the midpoint of the inflection tangent upon the second cycle.

#### 2.2.2 Single-Crystal X-ray Diffraction Studies

Single-crystal X-ray diffraction data of cyclic carbonate (CGFuC) were collected at 110 K on a Bruker Venture X-ray (kappa geometry) diffractometer with a Cu-Iμs X-ray tube (Kα = 1.54178 Å). The data integration and reduction were processed with SAINT software.^25^ An absorption correction was applied using the SADABS program.^26^ Hydrogen atoms were placed at idealized positions and were refined by fixed isotropic displacement parameters. Anisotropic displacement parameters were employed for all non-hydrogen atoms. The structure was solved and refined by the direct method using SHELXS/XT and was refined on *F* ^2^ by the full-matrix least-squares technique.^26^ The final pictorial presentation of structures was generated in the Olex2 software.^27^ Crystallographic data are deposited in the Cambridge Crystallographic Data Centre for CGFuC (CCDC-2477314).

#### 2.2.3 Mechanical Characterization

##### 2.2.3.1 Lap Shear Test

Stainless-steel substrates were mechanically abraded using sandpaper to introduce surface roughness and remove surface oxide layers. After cleaning with methanol and drying with a paper towel, polymer powders were placed between overlapping substrate areas with a rectangular aluminum mold (14.5 × 20.3 × 0.3 mm) and hot-pressed at 85 °C under 60 psi for 5 min. This process was repeated three times to ensure a consistent bonding. The lap joint area was approximately 294.35 mm^2^, with the dimensions of each stainless steel plate being 3 mm by 20 mm. After bonding, the samples were cooled to room temperature before lap shear testing at a constant displacement rate of 1 mm/min.

##### 2.2.3.2 Nanoindentation

Nanoindentation was carried out on a Bruker Hysitron Biosoft in situ Indenter with a 400 mm sapphire spherical tip under ambient conditions. A 3 mm diameter and 1 mm thickness circle-shaped specimen was hot-pressed in an aluminum frame and fixed to a glass slide for each experiment, and the tip was set to 2 mm from the surface. The tip approached the sample at 0.05 μm s^–1^ and was then loaded at 0.05 μm s^–1^ for 20 s until a total displacement of 1.5 μm, held for 120 s, and finally unloaded and retracted at 0.05 μm s^–1^. The elastic moduli were calculated based on the Hertzian model using the below equation, where d is the total indentation depth, *P* is the applied load, *E* is the elastic modulus, and v is Poisson’s ratio. The equation is fit by least-squares to the loading data.

$$P=\frac{4E}{3\left(1-v^{2}\right)}\surd R\delta^{3/2}$$

The hardness was calculated on the unloading segment according to the Oliver and Pharr method, using correction factors ε = 0.75 and β = 1 and the area function for a 200 μm radius conical probe using the TriboQ Analytical App from Bruker.

### 2.3 Synthesis of Glycidyl Furoate (GFu) Monomer

#### 2.3.1 Pathway A

To a stirred solution of glycidol (110 mmol) in dichloromethane (CH_2_Cl_2_), triethylamine (NEt_3_) was added under constant stirring at ice cold temperature under an N_2_ atmosphere followed by dropwise addition of furoyl chloride (100 mmol) to the stirring reaction mixture maintaining ice cold temperature (Scheme S1). After the complete addition of furoyl chloride, the reaction mixture was slowly allowed to come to room temperature and the reaction was stirred for 4 h. The completion of reaction was monitored by thin layer chromatography (TLC). The reaction mixture was thoroughly washed with deionized water and then dried over Na_2_SO_4_. The solvent was then evaporated in a rotary evaporator to obtain the crude product. The crude product was purified by silica gel column chromatography using hexane and ethyl acetate as an eluent. The pure monomer was obtained as a colorless liquid after vacuum distillation at 135 °C. Yield = 85%; ^1^H NMR (CDCl_3_, 400 MHz): δ (ppm) 7.60 (1H, dd, *J* = 2 Hz, 4 Hz), 7.25 (1H, dd, *J* = 2 Hz, 4 Hz), 6.53 (1H, dd, *J* = 2 Hz, 4 Hz), 4.63 (1H, dd, *J* = 12 Hz, 4 Hz), 4.17 (1H, dd, *J* = 2 Hz, 4 Hz), 3.30–3.34 (1H, m), 2.90 (1H, t, 4 Hz), 2.73 (1H, dd, *J* = 2 Hz, 4 Hz). ^13^C NMR (CDCl_3_, 100 MHz): δ (ppm): 158.1, 146.7, 144.0, 118.4, 111.9, 65.2, 49.2, 44.5. ESI-MS (*m*/*z*): [M + H]^+^ calcd for C_8_H_8_O_4_, 169.0495; found, 169.0493.

#### 2.3.2 Pathway B

To a stirred solution of 2-furoic acid (100 mmol) in water was added an aqueous solution of sodium hydroxide (120 mmol) (NaOH) dropwise with vigorous stirring at room temperature for 2 h (Scheme S2). After 2 h, water was evaporated from the reaction mixture. The solid was dried thoroughly to obtain sodium salt of 2-furoic acid (FuANa). To the prepared sodium salt of 2-furoic acid, epichlorohydrin (720 mmol) was added and the reaction was stirred for 4 h at 80 °C. After 4 h, tetrabutyl ammonium bromide (TBAB) (0.908 mmol) was added to the reaction mixture, and the reaction was stirred for an additional 30 h at 80 °C. Then the reaction mixture was cooled to room temperature and centrifuged to remove unreacted FuANa. Further unreacted epichlorohydrin was removed by a rotary evaporator, and pure glycidyl furoate was obtained as a colorless liquid (yield = 70%) after vacuum distillation. The spectroscopic characterization of the compound matched with the values obtained when furoyl chloride was used for the preparation.

### 2.4 Procedure for Copolymerization of Glycidyl Furoate with CO_2_

In a glovebox, the catalyst, cocatalyst, GFu monomer, and 1.0 mL of solvent (CH_2_Cl_2_/toluene, 1:1 v/v) were placed in a 15 mL stainless steel reactor in an argon atmosphere (Scheme S3). All reactions were performed on the scale of 0.0040 mmol of catalyst, using ratios of 1/0.5/*X* for catalyst/cocatalyst/monomer where the molar ratio of epoxy monomer varied with different experiments. The reactor was pressurized to 0.5–3.0 MPa by CO_2_, and then the reaction mixture was stirred at room temperature for the allotted time. Then excess CO_2_ was vented, and a small amount of the resultant mixture was removed from the reactor for ^1^H NMR analysis to determine the selectivity and conversion. The polymeric material was purified by precipitation in methanol. Initial precipitation was performed by adding dichloromethane solution of polymer sample dropwise to acidic methanolic solution (4 drops of conc. HCl/50 mL MeOH), followed by two precipitations in methanol. The resulting polymer was subsequently dried under vacuum before further characterization.

### 2.5 Procedure for Cyclic Product Formation

In a 15 mL stainless steel reactor, the catalyst, cocatalyst, GFu monomer, and 1.0 mL of solvent (CH_2_Cl_2_/toluene, 1:1 v/v) were placed inside the glovebox in an argon atmosphere (Scheme S4). The reaction was done using a ratio of 250/1/2 for monomer/catalyst/cocatalyst with 0.0040 mmol of catalyst. The reactor was pressurized to 3.0 MPa with CO_2_ and stirred at room temperature for the allotted time. After the desired time, the excess CO_2_ gas was vented out and conversion to cyclic product was determined by ^1^H NMR analysis. The residue was purified by silica gel chromatography using ethyl acetate and hexane as an eluent to give a cyclic product as a white solid. Yield = 70%; ^1^H NMR (CDCl_3_, 400 MHz): δ (ppm) 7.61 (1H, d, *J* = 4 Hz), 7.25 (1H, d, *J* = 4 Hz), 6.52 (1H, dd, *J* = 2 Hz, 4 Hz), 4.99 (1H, m), 4.49–4.62 (2H, m), 4.49–4.62 (2H, m). ^13^C NMR (CDCl_3_, 100 MHz): δ (ppm): 157.8, 154.2, 147.3, 143.2, 119.4, 112.2, 73.7, 66.0, 63.3. ESI-MS (*m*/*z*): [M + H]^+^ calcd for C_9_H_8_O_6_, 213.0394; found, 213.0392.

### 2.6 Procedure for Copolymerization of GFu with COS

In a glovebox, the Cr-catalyst, PPNCl (cocatalyst), GFu monomer, and 1 mL of solvent (CH_2_Cl_2_/toluene, 1:1 v/v) were placed in a 15 mL stainless steel reactor vessel (Scheme S5). All reactions were performed on the scale of 0.0040 mmol of catalyst, using ratios of 1/0.5/*X* for catalyst/cocatalyst/monomer, where *X* = 250, 500, and 750 equiv. The reactor was pressurized to 1.0 MPa by COS and then the reaction mixture was stirred at room temperature. After 24 h, excess COS was discharged and a small amount of the resultant mixture was taken from the reactor for ^1^H NMR analysis. The polymer was purified by precipitation in methanol. Initial purification was performed by adding dichloromethane solution of polymer sample dropwise to acidic methanol solution (4 drops of conc. HCl/50 mL MeOH), followed by two precipitations in methanol. The polymer was subsequently dried in vacuum before further characterization.

## 3 Results and Discussion

### 3.1 Synthesis and Characterization of Glycidyl Furoate (GFu)

The monomer glycidyl furoate (GFu) was synthesized (Scheme 1) via a modified procedure as reported in the literature.^19,20^ In pathway A, the reaction involved a direct reaction of furoyl chloride and glycidol, whereas via pathway B, furoic acid was first converted to sodium furoate, followed by reaction with epichlorohydrin in the presence of tetrabutylammonium bromide (TBAB). The detailed synthetic methods for the synthesis are discussed in the Supporting Information.

**Scheme 1:**
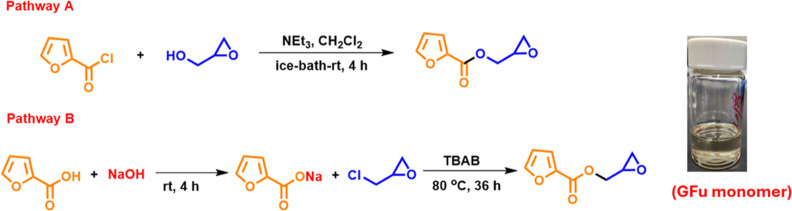
Synthetic Pathways for Glycidyl Furoate (GFu)

The epoxide was isolated as a colorless liquid at ambient temperature and characterized by spectroscopic techniques (NMR, FTIR, and mass spectrometry; Figures S1–S5). The ^1^H NMR spectrum of GFu monomer showed that the methylene proton (attached to the hydroxy group in glycidol and chloro group in epichlorohydrin) signal was shifted downfield (δ = 4.1–4.6 ppm) due to the formation of an ester linkage as compared to the chemical shift in glycidol and epichlorohydrin (δ = 3.4–3.8 ppm). Further, the formation of the ester linkage was confirmed by ^13^C NMR and IR studies. The signal for carbonyl carbon appeared at 158.1 ppm in the ^13^C NMR and the IR stretching frequency of C=O was observed at 1728 cm^–1^, characteristic of the ester group in the GFu monomer. The hydrogen adduct of the GFu monomer (*m*/*z* = 169.0493) was detected in the mass spectrometric analysis, which matched the simulated value.

### 3.2 Copolymerization of GFu Monomer with CO_2_

The cobalt complexes (Figure 3) employed as catalysts for the study were synthesized according to the reported procedure,^28^ and the chromium complex was purchased commercially. We started our experimental investigation for the ROCOP of the GFu monomer and CO_2_ by employing a binary catalytic system comprising Co­(salen)­TFA/PPNTFA in toluene/CH_2_Cl_2_ at ambient temperature and 3.0 MPa CO_2_ pressure (Scheme 2). In the past, we have shown these binary catalysts to be very effective at performing these copolymerization reactions under mild reaction conditions.

**Figure 3:**
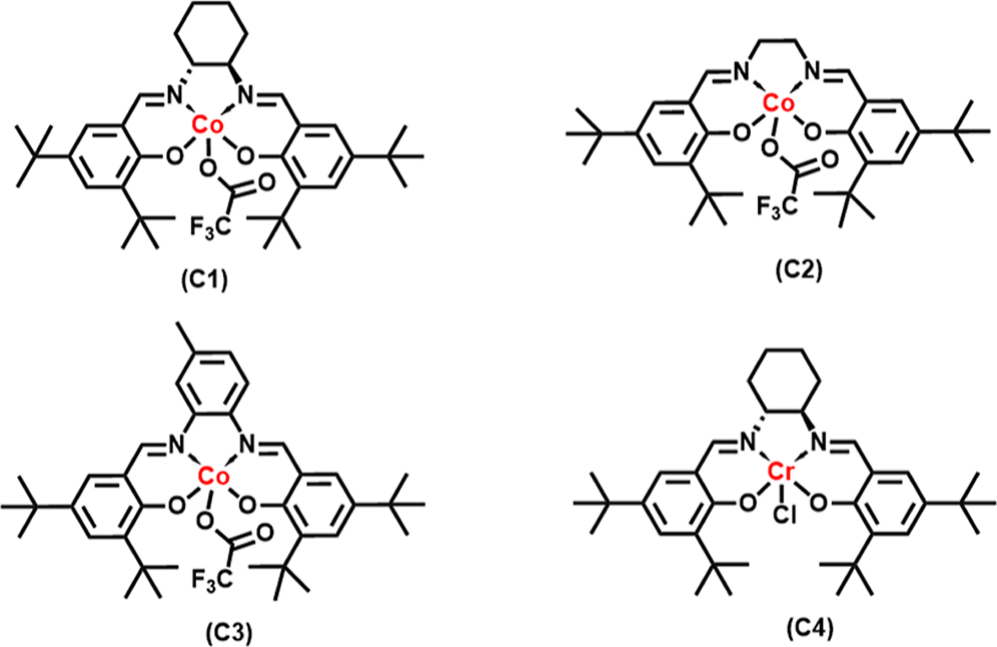
Co­(III) and Cr­(III) complexes as catalysts for the copolymerization reaction studies.

**Scheme 2:**
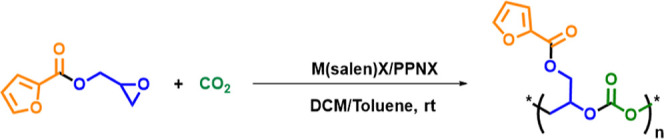
Copolymerization of GFu with CO_2_

Table 1 summarizes the results of all of the experimental investigations related to copolymerization studies of GFu with CO_2_. The reaction was first carried out for 24 h (Table 1, entry 1). The IR spectrum (Figures 4 and S6) of the reaction mixture gave us a qualitative idea of the formation of poly­(glycidyl furoate) carbonate (PGFuC) and cyclic carbonate (CGFuC) in the reaction medium. The two new characteristic bands were observed at 1757 cm^–1^ and 1818 cm^–1^, corresponding to the carbonate linkage in PGFuC and CGFuC, respectively. Furthermore, the ^1^H NMR spectrum showed a significant downfield shift in the methine proton signal from δ = 3.20 ppm (for free monomer) to δ = 5.20 ppm (for PGFuC) and δ = 4.99 ppm (for CGFuC) due to the formation of the carbonate linkage (Figure 5). The position of the methine signal due to PGFuC and CGFuC in the reaction mixture can be identified by comparing their peaks in their pure form (Figure 5). The ^1^H NMR spectrum of the crude reaction mixture revealed that after 24 h, 91% conversion was recorded for the monomer. The proportion of linear chain polycarbonate (PGFuC) was 85% and that of cyclic carbonate (CGFuC) was 15% (Figure S7). The reaction time was then increased to 36 h, and it was observed that on increasing the reaction time, there was almost complete conversion of monomer (Table 1, entry 2). Thereafter, further reactions were performed for 36 h under reaction conditions varying the catalyst, cocatalyst, cocatalyst loading, solvent, and CO_2_ pressure (Figure 6).

**Table 1:** ROCOP of GFu Monomer with CO_2_ at Ambient Temperature^a^

					selectivity				
entry	catalyst	cocat.	cat/cocat.	conv. (%)	PC	CC	carbonate linkages	*M*_n_ [kg mol^–1^]	*Đ*
1^b^	C1	PPNTFA	1/1	91	85	15	>99	16.6	1.13
2	C1	PPNTFA	1/1	98	81	19	>99	17.0	1.21
3	C1	PPNTFA	1/0.5	98	88	12	>99	22.1	1.29
4	C2	PPNTFA	1/0.5	92	82	18	>99	16.6	1.24
5	C3	PPNTFA	1/0.5	97	92	8	>99	19.0	1.20
6	C1	PPNN_3_	1/0.5	94	88	12	>99	20.9	1.21
7	C1	PPNCl	1/0.5	99	94	6	>99	22.5	1.24
8^c^	C1	PPNTFA	1/0.5	99	83	17	>99	27.2	1.20
9^d^	C1	PPNTFA	1/0.5	99	91	9	>99	14.5	1.17
10	C1	PPNTFA	1/2.0	99	38	62	>99	6.2	1.34
11	–	PPNTFA	0/0.5	–	–	–	–	–	–
12	–	PPNTFA	0/2.0	–	–	–	–	–	–
13	C4	PPNCl	1/2.0	99	0	>99	–	–	–
14^e^	C1	PPNTFA	1/0.5	94	66	34	>99	14.0	1.19
15^f^	C1	PPNTFA	1/0.5	96	78	22	>99	20.0	1.22
16^g^	C1	PPNTFA	1/0.5	98	80	20	>99	2.13	1.03

a Reactions were performed on the scale of 0.0040 mmol of catalyst, using ratios of 1/*x*/250 for catalyst/cocatalyst/monomer; solvent (1:1, CH_2_Cl_2_/toluene; 1 mL) in a 15 mL stainless steel reactor under 3.0 MPa of CO_2_ pressure for 36 h at ambient temperature.

b Reaction was performed for 24 h.

c 500 equiv of GFu used.

d Propylene carbonate was used as a solvent.

e Reaction performed under a 0.5 MPa CO_2_ pressure.

f Reaction performed under a 1.5 MPa CO_2_ pressure.

g Reaction performed with 7 equiv of terephthalic acid as a chain-transfer agent. PC = polycarbonate and CC = cyclic carbonate.

**Figure 4:**
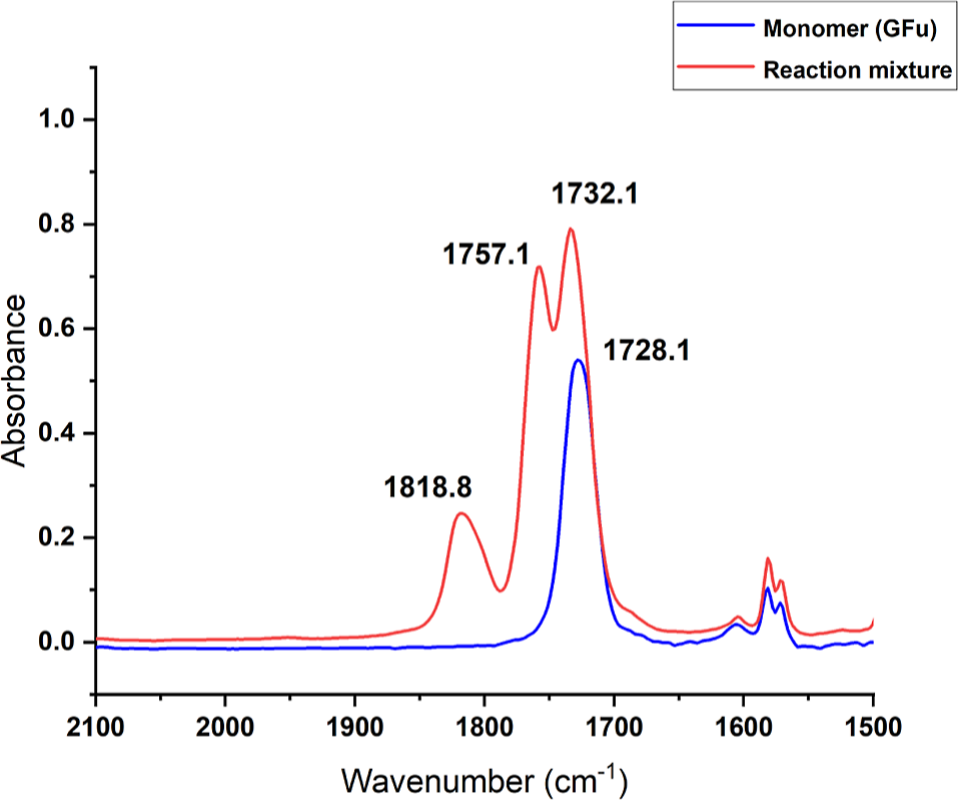
Stacked IR plot for a pure monomer and crude reaction mixture.

**Figure 5:**
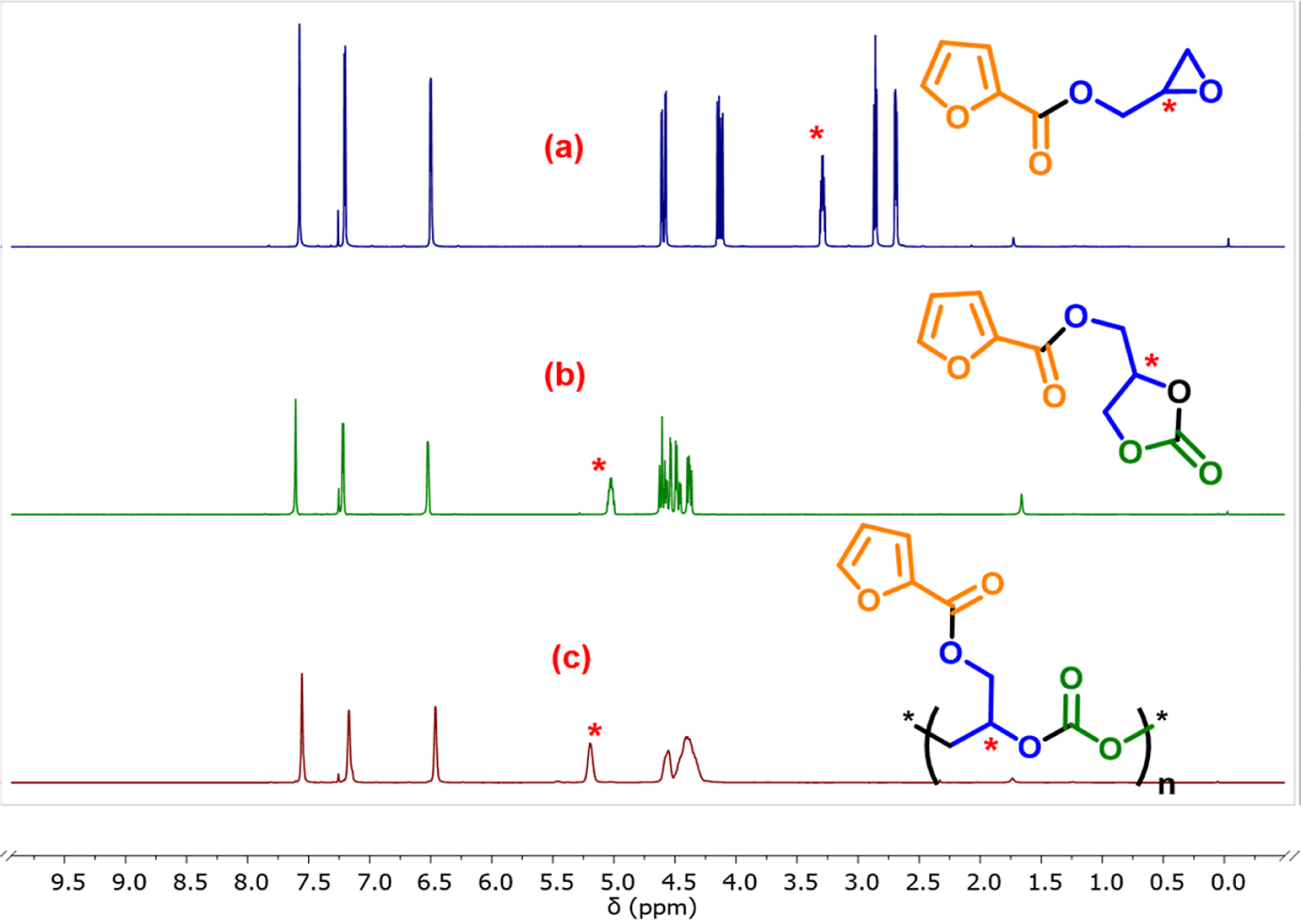
Stacked ^1^H NMR plots for (a) monomer (GFu); (b) CGFuC; (c) PGFuC.

**Figure 6:**
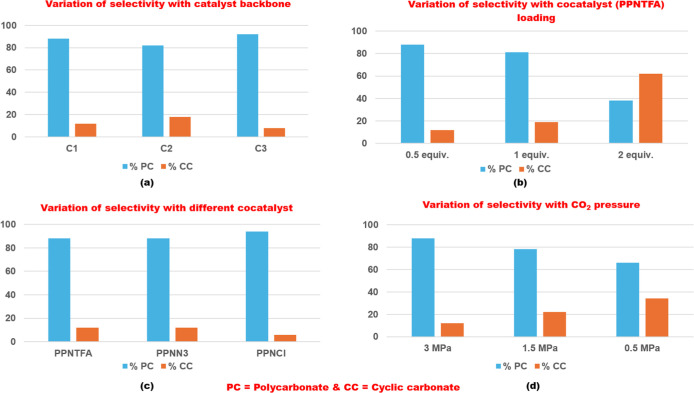
Comparison of product selectivity with (a) catalyst backbone, (b) cocatalyst loading, (c) nature of cocatalyst, and (d) CO_2_ pressure.

Next, the copolymerization reaction was carried out by reducing the amount of cocatalyst to 0.5 equiv (Table 1, entry 3). It was observed that on reducing the number of equivalents of cocatalyst, there was an improvement in the selectivity for the formation of polycarbonate (PGFuC = 88%) over cyclic carbonate (CGFuC = 12%), and there was a notable increase in the molecular weight of the polymer. The subsequent experiments were carried out to examine the effect of the variation in the catalyst backbone on the activity and selectivity for the copolymerization reactions (Table 1, entries 3–5). The three catalysts differed in the imine backbone of the salen ligand due to different diamines (cyclohexyldiamine, ethylenediamine, and 4-methyl-o-phenylenediamine) involved in ligand preparation. It was observed that the activity for the copolymerization reaction decreased when the cyclohexyl backbone was replaced with the ethylene backbone (Table 1, entries 3–4). A plausible reason for such an observation is that as the polymer chain grows, the steric crowding around the metal center increases; due to the flexible nature of the cyclohexyl ring, the consecutive epoxide coordination to the metal center is easier. The selectivity improved when an aromatic backbone replaced the cyclohexyl backbone (Table 1, entries 3 and 5). This can be explained by considering the electronic nature of the two backbones. The cyclohexyl backbone has an electron-donating nature, while the phenylene backbone has an electron-withdrawing nature. Due to the electron-withdrawing nature of the phenylene backbone, the Lewis acidity of the metal center increases. Thus, the growing polymer chain cannot easily detach from the metal center, leading to a decrease in the probability of backbiting reaction, and the percentage of cyclic product formation is less. We further studied the copolymerization reaction by using different phosphonium salts as cocatalysts (Table 1, entries 3, 6, and 7). It was observed that on replacing the anion of phosphonium salt from trifluoroacetate (PPPTFA) to azide (PPNN_3_), there was no change in selectivity, and there was a slight decrease in the conversion of monomer and molecular weight of the polymer (Table 1, entries 3 and 6). When PPNCl was used as a cocatalyst for the reaction, there was an improvement in selectivity for the reaction, while the molecular weight of the obtained polymers was similar (Table 1, entries 3 and 7). The formation of the cyclic product was minimal when PPNCl was used as a cocatalyst. Since chloride is a poor leaving group than trifluoroacetate, the formation of the cyclic product toward the beginning of the copolymerization reaction via intermolecular cyclic elimination is minimal. We then observed that on increasing the monomer loading from 250 to 500 equiv, there was not much of a significant increase in the molecular weight of the polymer (Table 1, entries 3 and 8), while there was a decrease in selectivity. Next, the copolymerization reaction was performed using the green and sustainable solvent propylene carbonate (PC). On using propylene carbonate as a reaction medium, there was a slight improvement in the selectivity for the formation of polycarbonate over cyclic carbonate, but the molecular weight of the polymer decreased (Table 1, entries 3 and 9). We then performed a subsequent set of experiments to establish the reaction conditions under which the copolymerization reaction results in the exclusive formation of cyclic carbonate (Table 1, entries 10–13). We observed that on increasing the cocatalyst loading to two equivalents, there was a significant increase in the percentage of cyclic carbonate formation over polycarbonate formation (Table 1, entries 2 and 10). Due to the increased cocatalyst loading, more nucleophiles are available in the reaction medium, which can easily displace the growing polymer chain from the metal center. The alkoxide end of the detached polymer chain can easily attack the adjacent carbonyl moiety, leading to backbiting reaction responsible for the formation of cyclic carbonate. The formation of cyclic carbonate is not possible by using cocatalyst alone. This was confirmed when reactions were performed in the absence of a catalyst at two different cocatalyst loadings (0.5 and 2.0 equiv) (Table 1, entries 11 and 12). It was observed that the monomer remains unreacted, indicating that the formation of the cyclic product is a result from backbiting reactions and cannot be solely initiated by the cocatalyst. The exclusive cyclic carbonate formation could be achieved by using C4 and PPNCl as a cocatalyst (Table 1, entry 13). Thereafter, the copolymerization reactions were performed at reduced CO_2_ pressure (0.5 and 1.5 MPa) (Table 1, entries 14–15). It was observed that on reducing the CO_2_ pressure, there was no significant change in activity but there was a substantial decrease in selectivity. Due to a decrease in CO_2_ pressure, there is a lower concentration of CO_2_ for insertion, into the growing polymer chain, and thus, the backbiting reaction becomes more feasible.

All of the crude copolymer samples obtained by the above experimental investigations were purified, and the pure products were characterized by spectroscopic techniques (NMR and IR) and gel permeation chromatography. All the characterization related to one copolymer sample is added in the Supporting Information (Figures S8–S11). The percentage selectivity in the polycarbonate linkage (>99%) was confirmed by ^1^H spectroscopy by the absence of any peak at δ = 3.4–3.5 ppm (signal due to the polyether linkage in the polymer chain) (Figure 7). The ^13^C NMR spectrum showed that the polymer chain showed an almost exclusive HT linkage (δ = 153.9 ppm) with a minor fraction of TT (δ = 154.3 ppm) and HH linkage (δ = 153.5 ppm) (Figure 8). The regioselectivity of the copolymers was further confirmed by performing a polymerization reaction with ^13^CO_2_. The ^13^CO_2_ NMR spectrum (Figure S9) showed the formation of HT regioisomers in a major proportion with a minor fraction of HH and TT regioisomers. The GPC traces (Figures S12–S18) of all the isolated copolymer samples exhibiting bimodal molecular weight distribution (Figure 9) may be attributed to the presence of adventitious moisture in the reaction medium.

**Figure 7:**
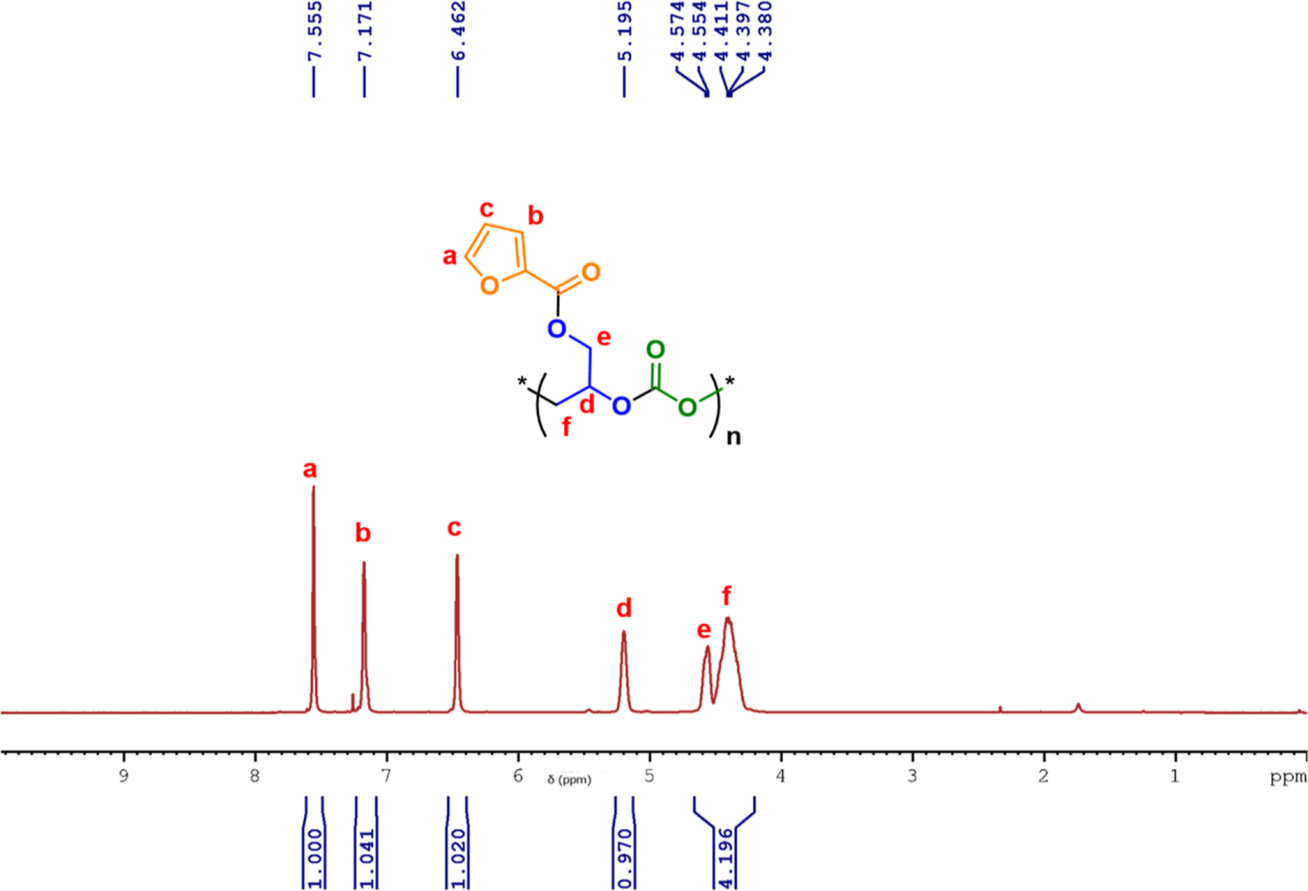
^1^H NMR spectrum (400 MHz, CDCl_3_) of poly­(glycidyl furoate) carbonate (PGFuC) with carbonyl region expanded in overlay.

**Figure 8:**
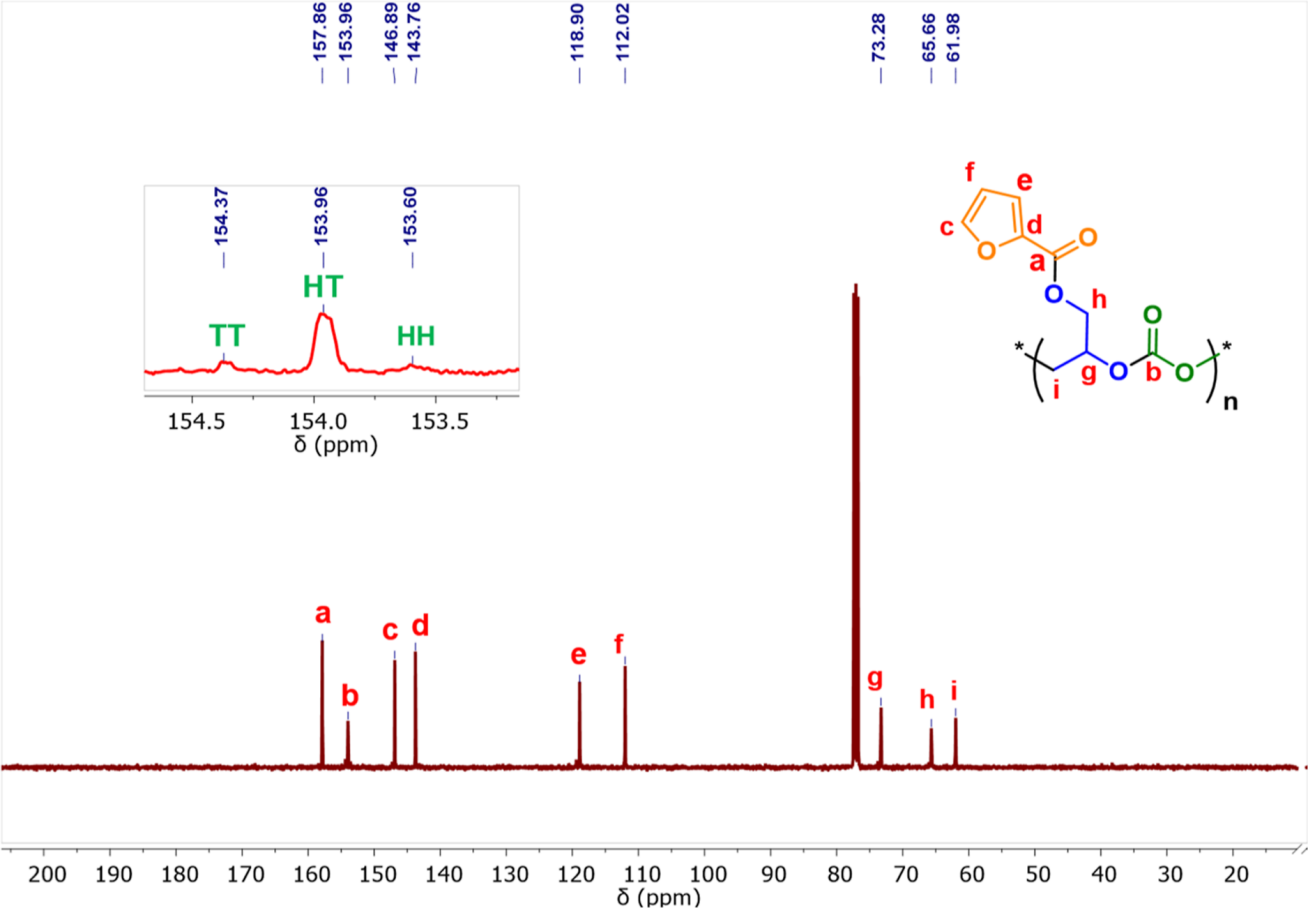
^13^C NMR spectrum (100 MHz, CDCl_3_) of poly­(glycidyl furoate) carbonate (PGFuC).

**Figure 9:**
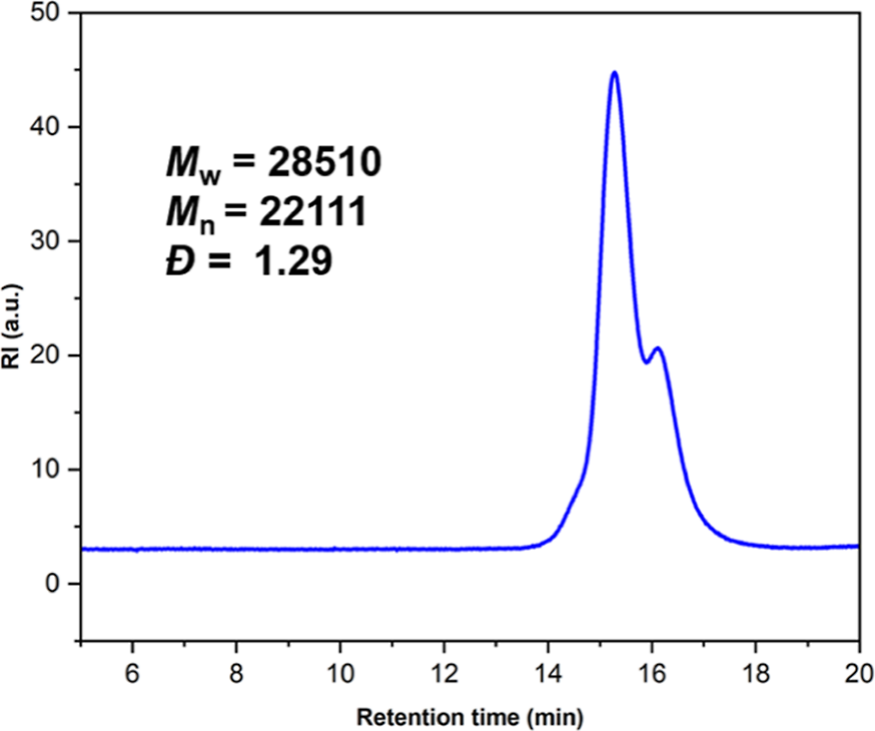
GPC trace of polycarbonate sample with catalyst 1 and 0.5 equiv of cocatalyst after 36 h (Table 1, entry 3).

An observation discernible from this work deserves specific comment. That is, the coupling of two closely related furfural derivatives GFu (a) and CFGE (b)^29^ with carbon dioxide under identical catalytic conditions provides strikingly different product selectivity, copolymer, and cyclic carbonate, respectively (Figure 10). Our suggestion at this point for this behavior is that GFu is more electron-donating, thereby being more activated for ring-opening followed by resulting enhancement for CO_2_ insertion.^30^ This outcome further supports the subtle sensitivity noted for product selectivity resulting from the coupling of CO_2_ and epoxides on both the catalyst and epoxide.

**Figure 10:**
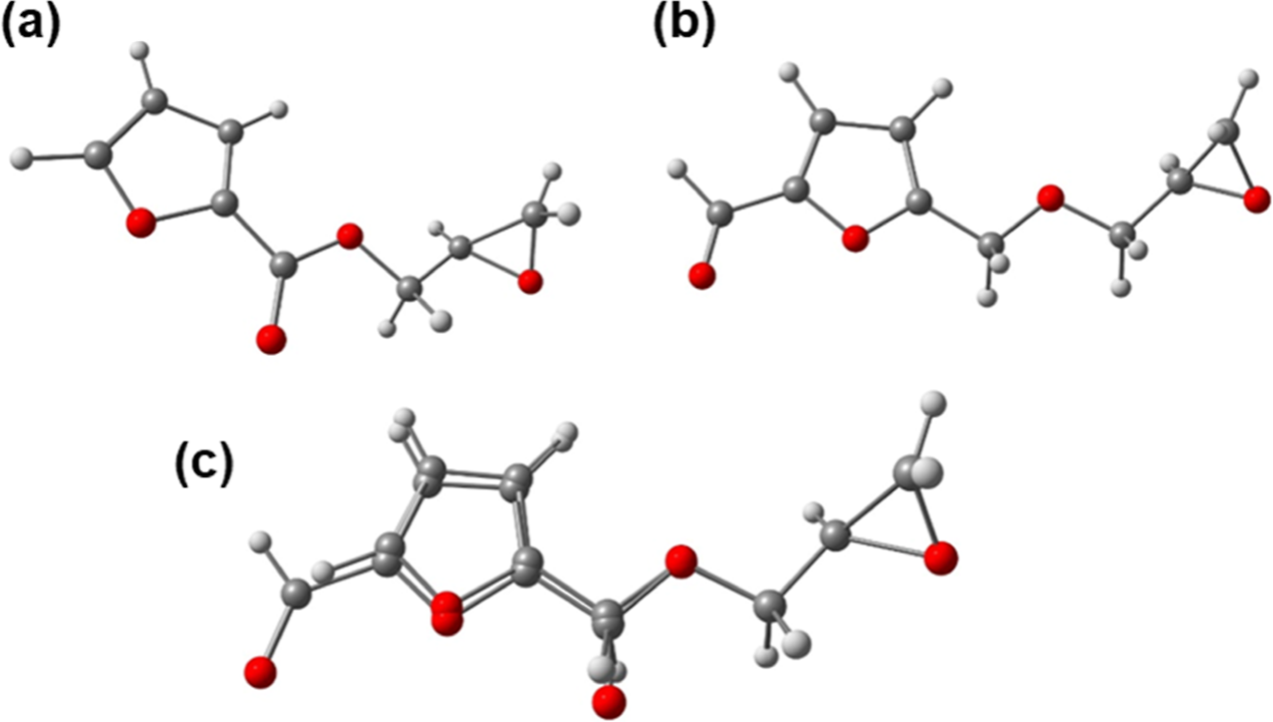
Epoxides (a) GFu and (b) CFGE and overlay in (c) with GFu in the bottom.

The cyclic carbonate (CGFuC) was characterized by different spectroscopic studies (Figures S19–S22). The IR stretching frequency of the carbonyl group (C=O) of cyclic carbonate was observed at 1818 cm^–1^ and the ^13^C NMR signal for carbonyl carbon was a sharp singlet at δ = 154.5 ppm. The mass of hydrogen adduct of CGFuC was found to be 213.0392. The structure of the cyclic carbonate was confirmed by single-crystal X-ray diffraction analysis (Figure 11). The colorless needle-shaped single crystals of CGFuC were grown by the slow vapor diffusion of hexane into a toluene/CH_2_Cl_2_ (1:1) solution of compound CGFuC at room temperature. The cyclic carbonate crystallizes in a triclinic crystal system with a *P* 1̅ space group. The shortest intermolecular distance is 3.625 Å, and it shows a weak C–H···O bonding interaction. The molecular structure and molecular packing diagram of CGFuC are shown in Figures 10 and S23, respectively. The crystallographic data and data collection parameters are provided in Table S1. The bond distance and bond angle parameters of CGFuC are given in Tables S2 and S3, respectively. The XRD structure shows that the C=O bond distance of the carbonate group is shorter (1.190 Å) than the C=O bond distance in the ester group (1.205 Å).

**Figure 11:**
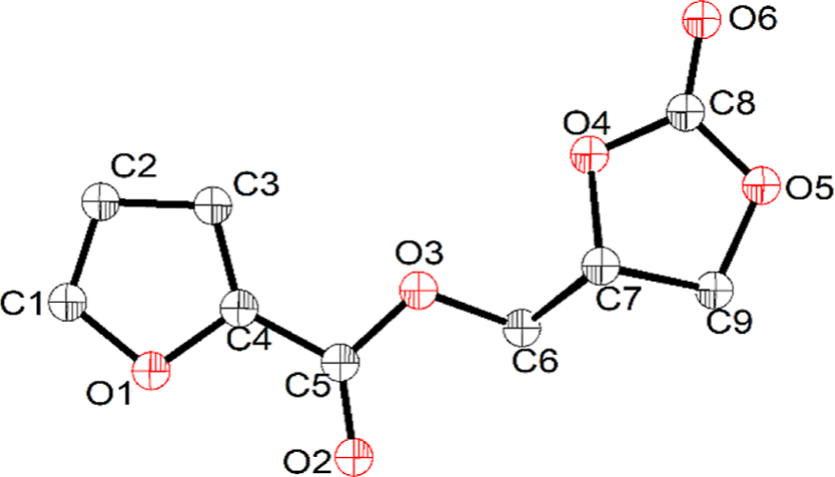
Molecular structure of CGFuC (with 40% probability of ellipsoid) at 110 K.

The low molecular weight polymer was prepared for MALDI-TOF analysis in the presence of terephthalic acid as a chain transfer agent (Table 1, entry 16). The oligomer showed a unimolecular molecular weight distribution, as observed in the GPC trace (Figure 12a). The MALDI-TOF spectrum of the oligomer exhibited two series of peaks (Figure 12b). The major series of peaks corresponds to the {M­(CTA + GFu) + nM­(GFu + CO_2_) + M­(GFu) + M­(Na^+^)} and the minor series of peaks corresponds to {M­(CTA + GFu) + mM­(GFu + CO_2_) + M­(H) + M­(K^+^)}. The mass difference between the two consecutive members of each series was found to be 212u, which is the total mass of (GFu + CO_2_) taken together, corresponding to the repeating unit in the polymer chain.

**Figure 12:**
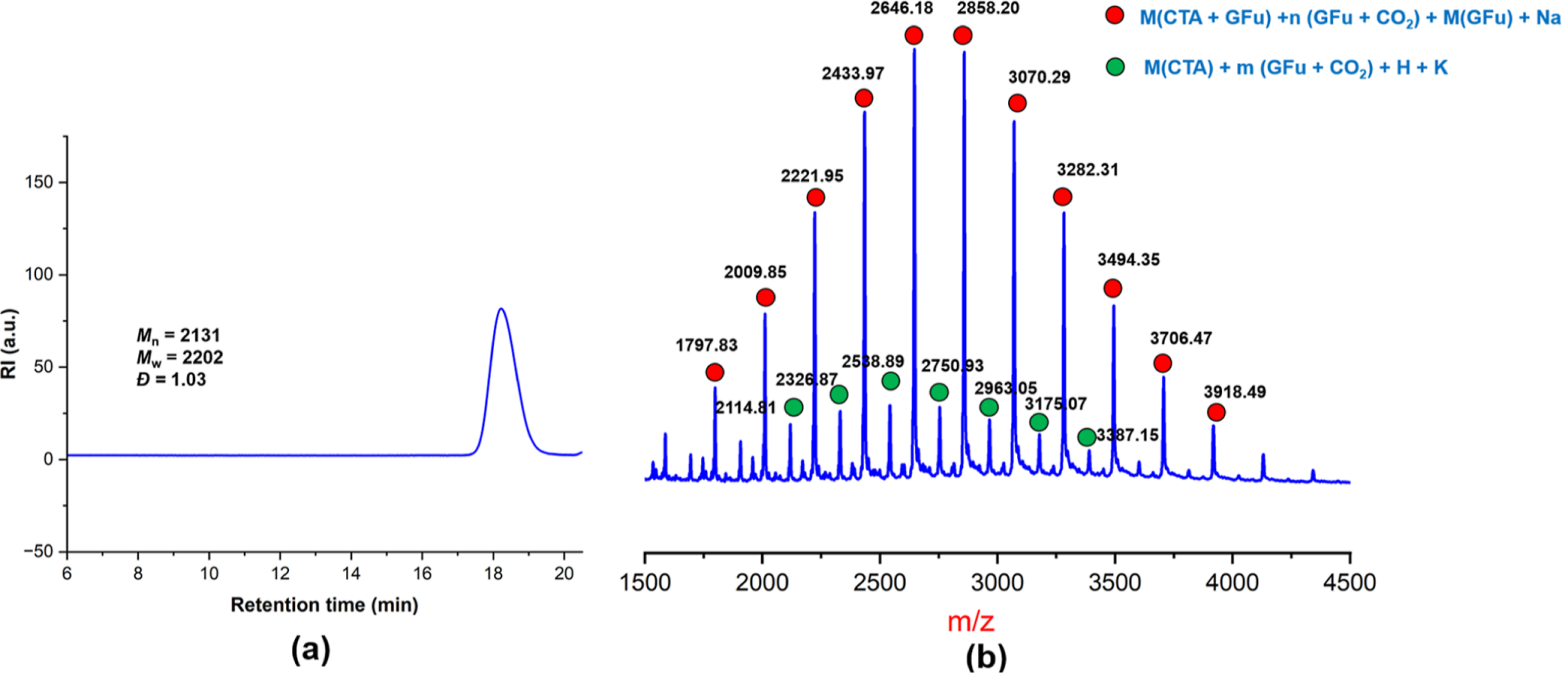
(a) GPC trace of oligomer of GFu/CO_2_ copolymer. (b) MALDI-TOF spectrum of GFu/CO_2_ copolymer.

### 3.3 Copolymerization of GFu Monomer with COS

We further investigated the ROCOP of the GFu monomer with COS to synthesize sulfur-containing polymers. The existing literature reported to date for the formation of poly­(monothiocarbonates) from ROCOP of epoxide with COS has centered around the binary catalyst (salen)­CrCl/onium salt catalyst.^31^ So, we chose (salen)­CrCl/PPNCl to screen the GFu monomer for copolymerization with COS (Scheme 3). Table 2 summarizes the experimental findings relating to the copolymerization studies of GFu monomer with COS. It was observed that (salen)­CrCl/PPNCl was the active catalyst for the copolymerization of GFu monomer with COS with excellent selectivity for the formation of poly­(monothiocarbonates) over cyclic thiocarbonate in each case as the monomer loading is varied from 250 to 750 equiv. The reaction time for the same monomer loading was less for the reaction of the GFu monomer with the more reactive COS than for CO_2_. It was observed that when monomer loading was varied from 250 to 500 equiv, the molecular weight of the polymer doubled; however, upon further increasing the monomer loading to 750 equiv, the increase in molecular weight was less. The formation of a thiocarbonate linkage in the polymer chain was confirmed by the downfield shift of the methine proton signal (δ = 3.20 ppm) for free monomer to (δ = 5.40 ppm) for PGFuTC (Figure S24). The carbonyl carbon signal in the ^13^C NMR spectrum for poly­(monothiocarbonate) was observed at δ = 169.5 ppm, indicative of no oxygen/sulfur scrambling taking place (Figure S25). The IR band for C=O of thiocarbonate was observed at 1730 cm^–1^, which was broad as it merged with the C=O stretching of the ester group (Figure S27). The GPC trace of all of the poly­(monothiocarbonate) samples was bimodal (Figure S28). The MALDI-TOF spectrum (Figure S29) for PGFuTC showed a fragmentation pattern corresponding to two series where the mass difference between consecutive members in the same series is *m*/*z* = 228 which is equal to the mass of one molecule of GFu monomer and one molecule of COS taken together. The major series corresponds to {M­(CTA + GFu) + nM­(GFu + CO_2_) + M­(GFu) + M­(Na^+^)} and the minor series corresponds to M­(Cl) + n­(GFu + CO_2_) + M­(GFu) + Na.

**Scheme 3:**
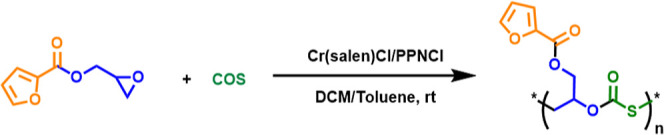
Copolymerization of GFu with COS

**Table 2:** ROCOP of GFu Monomer with COS at Ambient Temperature and 1.0 MPa of COS Pressure^a^

			selectivity				
entry	monomer/cat/cocat.	conv. (%)	PMTC	CMTC	thiocarbonate linkages (%)	*M*_n_ [kg mol^–1^]	*Đ*
1	250/1/0.5	>99	>99		>99	13.2	1.47
2	500/1/0.5	>99	>99		>99	26.9	1.33
3	750/1/0.5	>99	>99		>99	30.1	1.31

a Reactions were performed on the scale of 0.0040 mmol of catalyst, using ratios of 1/0.5/*x* for catalyst/cocatalyst/monomer; solvent (1:1, CH_2_Cl_2_/toluene; 1 mL) in a 15 mL stainless steel reactor for 24 h at ambient temperature.

### 3.4 Terpolymerization Studies

Consistent with the lack of copolymerization of GFu and COS in the presence of the Co­(III) catalyst system as well as the absence of copolymerization from GFu and CO_2_ utilizing the Cr­(III) catalyst (Table 1, entry 13), attempts at terpolymerization of GFu/CO_2_/COS with the binary catalyst were unsuccessful. In the former instance, the Co­(III) catalyst was unstable in the presence of sulfur-containing monomers, and in the latter case, the Cr­(III) catalyst afforded exclusively monothiocarbonate (Figures S30–S34).

### 3.5 Thermal and Mechanical Properties

The thermal properties of polycarbonate and poly­(monothiocarbonate) were studied by thermogravimetric analysis (TGA) and differential scanning calorimetry (DSC). The TGA traces revealed that both varieties of polymer are stable up to 150 °C (Figure S35). The DSC traces showed that polycarbonate sample exhibited a slightly high glass transition temperature (58.6 °C) compared to poly­(monothiocarbonate) sample (49.2 °C), as is commonly observed (Figure 13).

**Figure 13:**
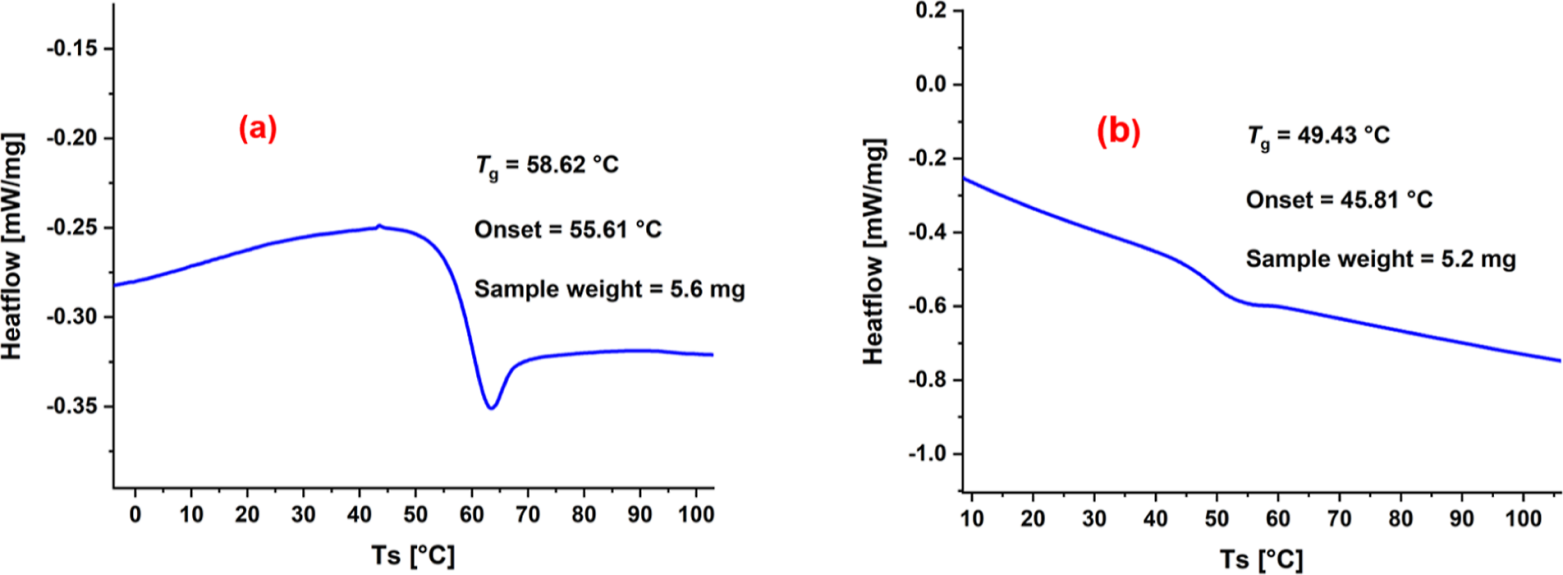
DSC trace for (a) polycarbonate and (b) poly­(monothiocarbonate).

The elastic modulus and hardness were determined by performing a nanoindentation test for the polymer sample.^32^ The experiment was done by pressing a spherical tip with a radius of 200 μm at a speed of 0.05 μm/s on the surface. The tip was held at a constant depth for 2 min before being retracted from the surface. The experiments were repeated twice, and the hardness and elastic modulus calculated by taking the average of the two values. Figure 14a shows the representative plot of load versus displacement, and Figure 14b shows the plot of load versus time for the polymer samples. Figure S36 shows samples prepared for the nanoindentation test. The polymer samples were transparent after hot pressing, as seen in Figure S36. The hardness and elastic modulus were found to be 4.39 GPa and 104.9 MPa for the polycarbonate sample and 4.90 GPa and 122.5 MPa for the poly­(monothiocarbonate) sample. The reason for slightly higher elastic modulus and hardness for poly­(monothiocarbonate) compared to polycarbonate may be attributed to the large and more polarizable sulfur atom, which results in increased intermolecular forces.

**Figure 14:**
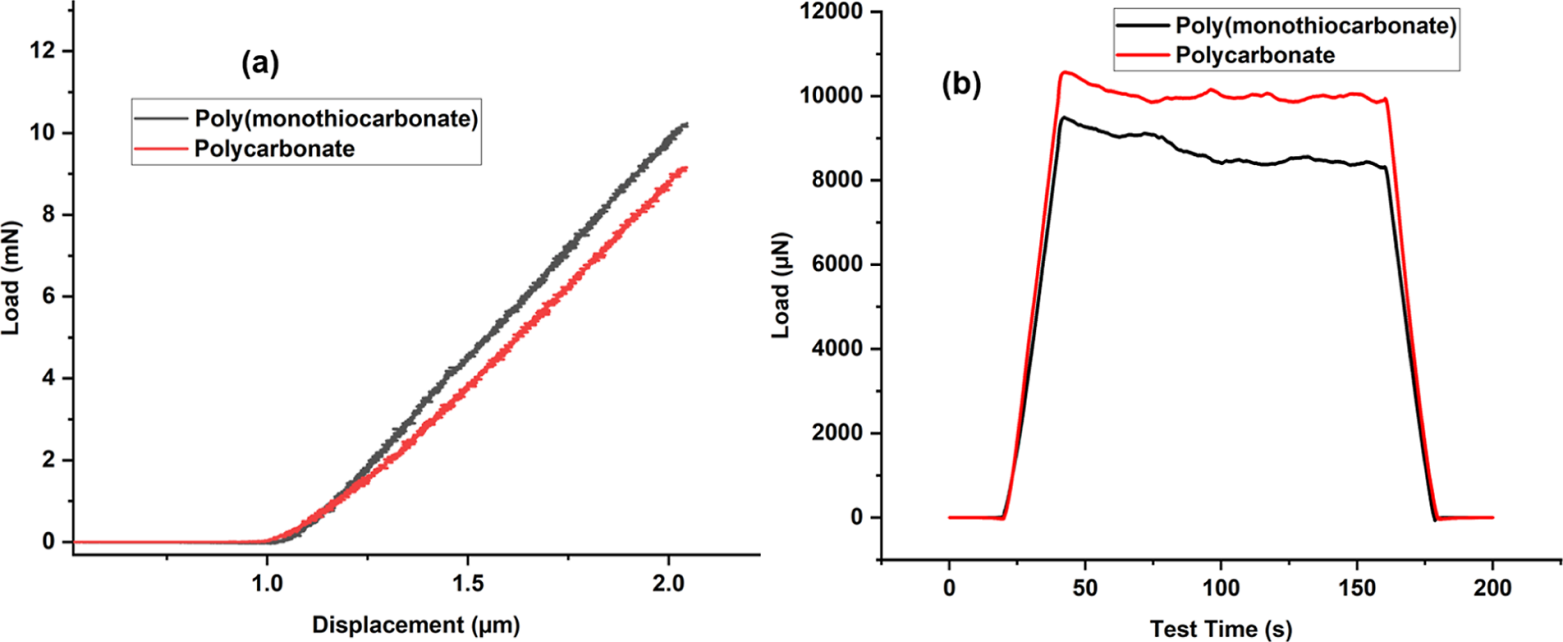
Representative plots for (a) load versus displacement. (b) Load versus time for polymer samples.

The adhesive behavior of aliphatic polycarbonate and poly­(monothiocarbonate) samples was assessed by performing a lap shear test^33^ using stainless steel as the substrate under identical conditions. Each of the polymer samples was put between stainless-steel plates and hot-pressed at 85 °C and 60 psi for 5 min, repeated three times to ensure uniform adhesion. The bonded plates were cooled to room temperature prior to testing. Figure S37 shows samples prepared for the lap shear test. The stress–displacement profiles (Figure 15) exhibited minimal differences in the slopes for the two samples. The poly­(monothiocarbonate) sample reached a maximum shear strength of 1.00 MPa before exhibiting abrupt interfacial failure, with minimal post-peak deformation. In contrast, the polycarbonate adhesive test did not fail within the displacement range of the instrument (ca. 50 mm), displaying continuous load-bearing capacity with increasing stress and extended plastic deformation. The findings suggested a higher cohesive and interfacial strength for polycarbonates compared to poly­(monothiocarbonates), although the exact failure point could not be determined due to instrument limitations. The difference in performance may be attributed to both bulk and interfacial factors. The higher polarity of the polycarbonate backbone, in combination with effective hydrogen bonding and better wetting on the oxidized stainless-steel surface, likely contributes to stronger adhesion. The presence of flexible C–S bonds and reduced polarity in the poly­(monothiocarbonate) may limit both cohesive strength and interfacial interactions, resulting in premature failure.

**Figure 15:**
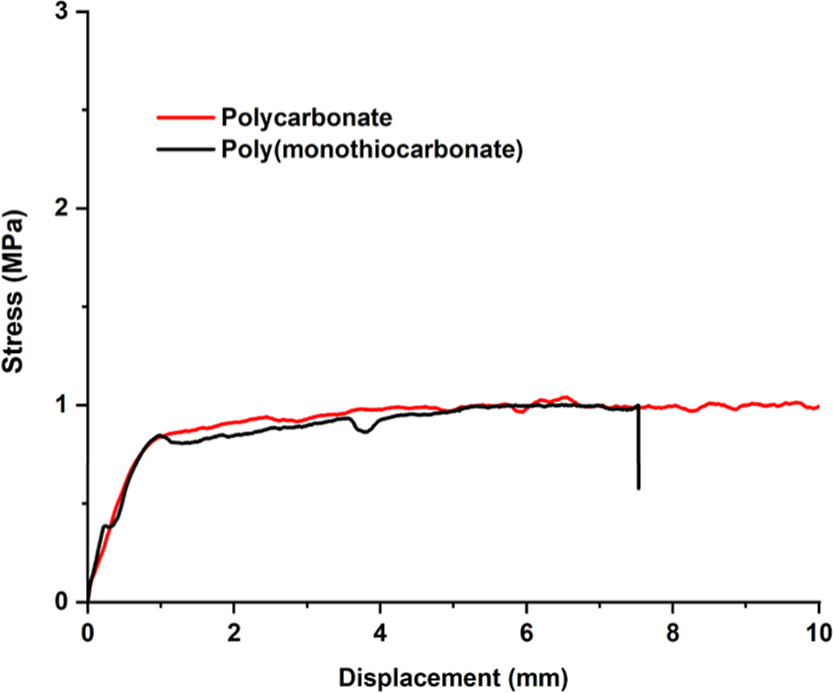
Lap shear test with polycarbonate and poly­(monothiocarbonate) sample.

The difference in performance may be attributed to both bulk and interfacial factors. The higher polarity of the polycarbonate backbone, in combination with effective hydrogen bonding and better wetting on the oxidized stainless-steel surface, likely contributes to stronger adhesion. The presence of flexible C–S bonds and reduced polarity in the poly­(monothiocarbonate) may limit both cohesive strength and interfacial interactions, resulting in premature failure.

### 3.6 Hydrolytic Degradation and Chemical Recycling

The degradation behavior of the polymers plays a crucial role in determining the life cycle of plastics and thus governs end-use applications. It is well-known in the literature that aliphatic polycarbonates generate alcohol and carbon dioxide when subjected to hydrolytic degradation.^34^ Therefore, we investigated the hydrolytic degradation behavior of polycarbonate and poly­(monothiocarbonate) under basic and acidic conditions. The preliminary hydrolytic degradation studies were performed in the presence of 1.0 (N) NaOH solution and 1.0 (N) HCl solution at room temperature for 30 min with polymer samples dissolved in THF. It was observed that in case of polycarbonate sample, the polymer degraded rapidly into a cyclic product and diol under basic conditions (Figure S38), while under acidic conditions, the polymer remained unaffected. The formation of the cyclic product along with diol indicates that the degradation reaction proceeds via backbiting of the deprotonated polymer chain and subsequent hydrolysis of the backbiting product. It was observed that when the reaction time was increased to 60 min, there was exclusive formation of diol (Figure S39). Furthermore, hydrolytic degradation of the poly­(monothiocarbonate) resulted in the formation of some fraction of cyclic product initially when the reaction was performed under basic conditions, while under acidic conditions, they remained unaffected. No complete conversion to a cyclic product or diol formation was noticed when poly­(monothiocarbonate) was hydrolyzed at elevated temperature (60 °C) for increased reaction time. Thus, we can say that poly­(monothiocarbonates) is hydrolytically much more stable than polycarbonates. Furthermore, we investigated the recyclability of the diol obtained from the hydrolysis of polycarbonate to give back epoxide monomer following the procedure reported recently by our research group.^35^ We were successful in getting back the epoxide monomer by the tosylation of diol (Figure S40) followed by ring-closing in the presence of base (Scheme 4).

**Scheme 4:**
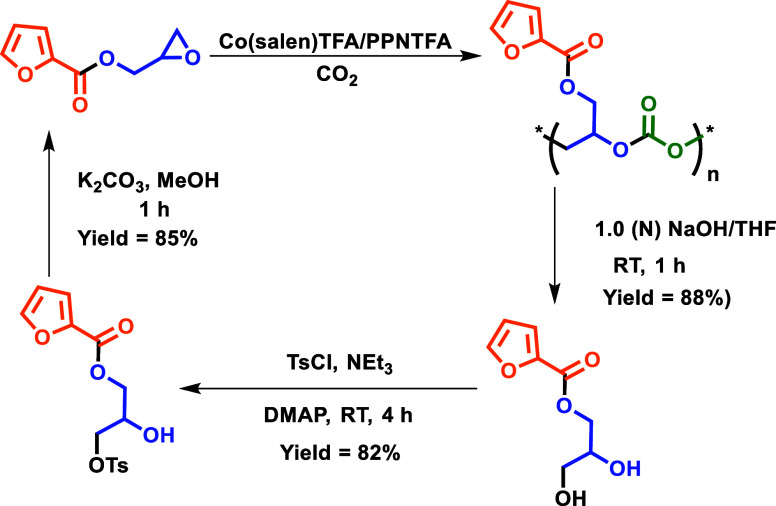
Hydrolytic Degradation and Chemical Recycling Back to Epoxide Monomer

## 4 Conclusions

The current study set out to investigate an epoxide derived from nonfood-based biomass as a comonomer for the ROCOP with two carbon-containing heteroallene gases (CO_2_ and COS). The epoxide under study was synthesized starting from commercially available furfural derivatives via a one-step (for furoyl chloride) and two-step (for furoic acid) synthetic strategy on a multigram scale with good yield. The furan-based epoxide monomer (GFu) is of interest, as furfural is a promising platform compound derived from lignocellulosic biomass. The copolymerization studies were conducted using binary catalyst systems comprising salen-Co/Cr complexes as catalysts and phosphonium salts as cocatalysts at ambient temperature in a 1:1 toluene/dichloromethane mixture. It was observed that GFu/CO_2_ copolymerization activity and selectivity were influenced by the imine linker of the salen backbone, nature of cocatalyst, cocatalyst loading, and CO_2_ pressure. The polycarbonate chain showed negligible polyether content (selectivity >99%) and exclusive formation of the HT linkage. The reaction was also successful when propylene carbonate was used as a solvent. The GFu monomer copolymerized with COS to produce aliphatic poly­(monothiocarbonate), adding to the limited list of epoxides that have been used to produce COS-derived copolymers. Both varieties of copolymer were thermally stable up to 150 °C with a glass transition value for the COS polymer slightly less than that of the CO_2_-based polymer. The polycarbonate sample exhibited better adhesive performance but less hardness and elastic modulus than poly­(monothiocarbonate). The polycarbonate sample was hydrolytically degraded to a diol under basic conditions. The diol generated was recycled back to the epoxide, making the entire polymerization/degradation process partially circular. The study prompts us to investigate further viable epoxides synthesized from furan derivatives as comonomers for ROCOP studies and to explore the potential of the GFu epoxide in synthesizing block polymers and its applications.

## Supplementary Material





## Notes

^1^H NMR, ^13^C NMR, and ATR FT-IR spectra of GFu monomer and corresponding CO_2_/COS polymers; GPC traces for polymers; and additional experimental details, materials, and methods (PDF)
Crystallographic data for C_9_H_8_O_6_ (CIF)
