# Supplementary material for: Gateway to Sustainable Polymers via Catalytic ROCOP of CO2/COS Utilizing a Renewable Epoxide Monomer from Furfural Derivatives
Source: ACS Sustain Chem Eng. 2025 Oct 8;13(43):18845–57. doi: 10.1021/acssuschemeng.5c08323 (PMC12587447; doi:10.1021/acssuschemeng.5c08323)
Supplement: Supplementary file 1 [file sc5c08323_si_001.pdf]

Supporting Information

Gateway to Sustainable Polymers via Catalytic ROCOP of CO_2/COS Utilizing a Renewable Epoxide Monomer from Furfural Derivatives

Sriparna Sarkar, [‡a] Mani Sengoden,[‡a] Chia-Min Hsieh[a], Peiran Wei[b], Sarnali Sanfui[a], Donald J. Darensbourg [*a]

‡ These authors contributed equally for this work.

[a] Department of Chemistry, Texas A&M University, College Station, Texas 77843, USA.

[b] Soft Matter Facility, Texas A&M University, College Station, TX 77843, USA.

*E-mail: djdarens@chem.tamu.edu

Number of pages: 31

Number of Figures: 40

Number of Schemes: 7

Number of Tables: 3

Contents

Scheme S1	Synthesis of glycidyl furoate (GFu) monomer via pathway A.	S5
Scheme S2	Synthesis of glycidyl furoate (GFu) monomer via pathway B.	S5
Figure S1	^{1}H NMR spectrum (400 MHz, $CDCl_3$) of glycidyl furoate (GFu).	S5
Figure S2	^{13}C NMR spectrum (100 MHz, $CDCl_3$) of glycidyl furoate (GFu).	S6
Figure S3	DEPT-135 spectrum of glycidyl furoate (GFu).	S6
Figure S4	ATR FT-IR of glycidyl furoate (GFu) in CH_2Cl_2.	S7
Figure S5	ESI-MS spectrum of glycidyl furoate (GFu).	S7
Scheme S3	Copolymerization of GFu with CO_2.	S8
Figure S6	ATR FT-IR spectrum of reaction mixture of GFu with CO_2 after 24 h.	S8
Figure S7	^{1}H NMR spectrum (400 MHz, $CDCl_3$) of reaction mixture of GFu with CO_2 after 24 h.	S9
Figure S8	^{1}H NMR spectrum (400 MHz, $CDCl_3$) of poly(glycidyl furoate) carbonate (PGFuC).	S10
Figure S9	^{13}C NMR spectrum (100 MHz, $CDCl_3$) of poly(glycidyl furoate) carbonate (PGFuC).	S10
Figure S10	DEPT-135 spectrum of poly(glycidyl furoate) carbonate (PGFuC).	S11
Figure S11	ATR FT-IR of poly (glycidyl furoate) carbonate (PGFuC) in CH_2Cl_2.	S11
Figure S12	GPC trace of polycarbonate sample with catalyst **1** after 24 h.	S12
Figure S13	GPC trace of polycarbonate sample with catalyst **1** after 36 h.	S12
Figure S14	GPC trace of polycarbonate sample with catalyst **2** & **3**.	S13
Figure S15	GPC trace of polycarbonate sample with catalyst **1** and different cocatalyst	S13
Figure S16	GPC trace of polycarbonate sample with increased loading of monomer.	S13
Figure S17	GPC trace of polycarbonate sample with propylene carbonate as solvent.	S14
Figure S18	GPC trace of polycarbonate sample at varying CO_2 pressure	S14
Scheme S4	Reaction of GFu with CO_2 to give cyclic carbonate.	S14
Figure S19	^{1}H NMR spectrum (400 MHz, $CDCl_3$) of cyclic (glycidyl furoate) carbonate (CGFuC).	S15
Figure S20	^{13}C NMR spectrum (100 MHz, $CDCl_3$) of cyclic (glycidyl furoate) carbonate (CGFuC).	S15

Figure S21	ATR FT-IR of cyclic (glycidyl furoate) carbonate (CGFuC) in CH_2Cl_2.	S16
Figure S22	ESI-MS spectrum of cyclic (glycidyl furoate)carbonate (CGFuC).	S16
Figure S23	Molecular packing structure of cyclic (glycidyl furoate)carbonate (CGFuC).	S17
Table S1	Crystal data and data collection parameters for CGFuC.	S18
Table S2	Bond distance parameters for CGFuC.	S19
Table S3	Bond angle parameters for CGFuC.	S20
Scheme S5	Copolymerization of GFu with COS	S21
Figure S24	^{1}H NMR spectrum (400 MHz, $CDCl_3$) of PGFuMTC.	S21
Figure S25	^{13}C NMR spectrum (100 MHz, $CDCl_3$) of PGFuMTC.	S22
Figure S26	DEPT-135 spectrum of PGFuMTC.	S22
Figure S27	ATR FT-IR of PGFuMTC.	S23
Figure S28	GPC trace of poly(monothiocarbonate) sample at three different monomer loading.	S24
Figure S29	GPC & MALDI trace of oligomer of GFu/COS copolymer.	S24
Figure S30	^{1}H NMR spectrum (400 MHz, $CDCl_3$) of reaction mixture of terpolymerization of GFu, COS and CO_2 with Co(III) catalyst.	S25
Figure S31	ATR FT-IR spectrum of reaction mixture of terpolymerization of GFu, COS and CO_2 with Co(III) catalyst.	S25
Figure S32	^{1}H NMR spectrum (400 MHz, $CDCl_3$) of reaction mixture of terpolymerization of GFu, COS and CO_2 with Cr(III) catalyst.	S26
Figure S33	^{13}C NMR spectrum (400 MHz, $CDCl_3$) of reaction mixture of terpolymerization of GFu, COS and CO_2 with Cr(III) catalyst.	S26
Figure S34	ATR FT-IR spectrum of reaction mixture of terpolymerization of GFu, COS and CO_2 with Cr(III) catalyst.	S27
Figure S35	TGA trace for Polycarbonate & Poly(monothiocarbonate).	S27
Figure S36	Samples prepared for nanoindentation test	S28
Figure S37	Samples prepared for lap shear test.	S28
	Procedure for polycarbonate degradation.	S28
Scheme S6	Polycarbonate hydrolysis to diol.	S29
Figure S38	^{1}H NMR spectrum (400 MHz, $CDCl_3$) of hydrolysis of PGFuC after 30 minutes.	S29
Figure S39	^{1}H NMR spectrum (400 MHz, $CDCl_3$) of hydrolysis of PGFuC after 60 minutes.	S30
		S30

	Procedure for preparation of glycidyl furoate epoxide (GFu) from diol.	
Scheme S7	Synthesis of glycidyl furoate epoxide (GFu) from diol.	S31
Figure S40	^{1}H NMR spectrum (400 MHz, $CDCl_3$) of tosylated product obtained from diol.	S31

Procedure for the preparation of glycidyl furoate (GFu) monomer

Pathway A

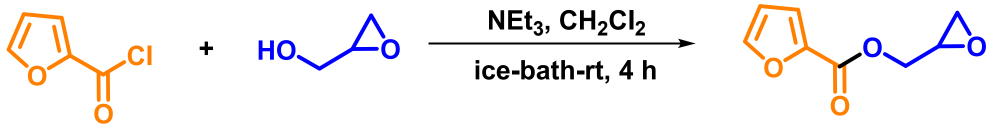

Scheme S1 Synthesis of glycidyl furoate (GFu) monomer via pathway A.

Pathway B

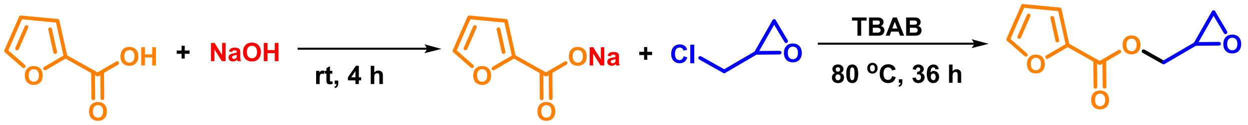

Scheme S2 Synthesis of glycidyl furoate (GFu) monomer via pathway B.

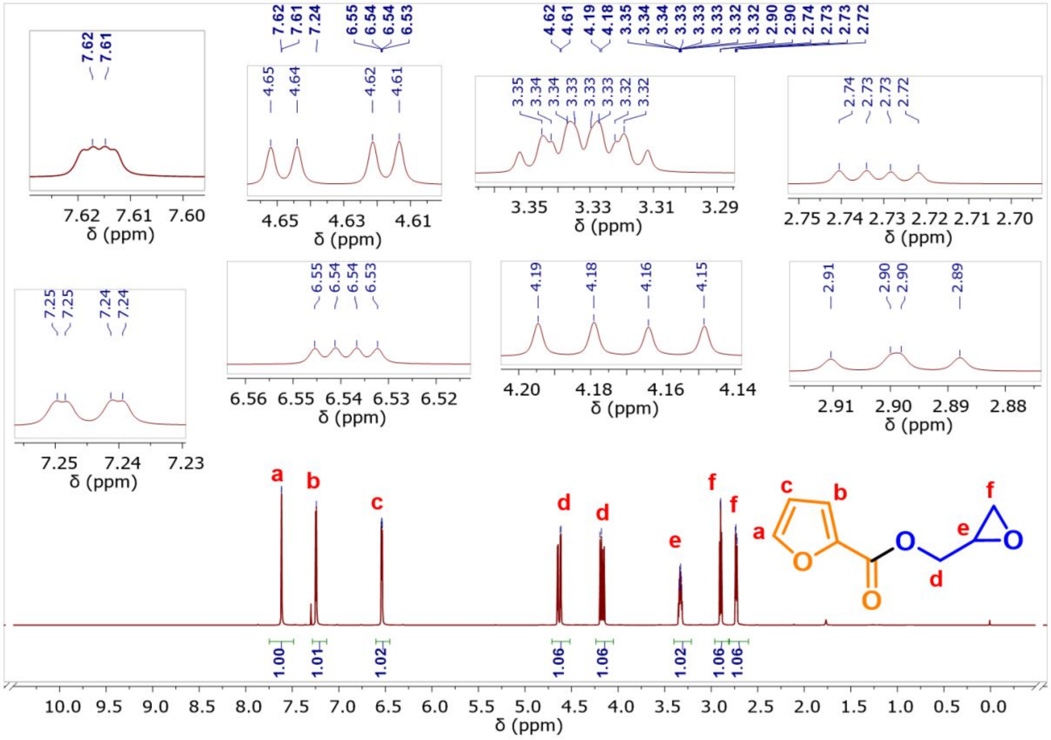

Figure S1: ^{1}H NMR spectrum (400 MHz, $CDCl_3$) of glycidyl furoate (GFu).

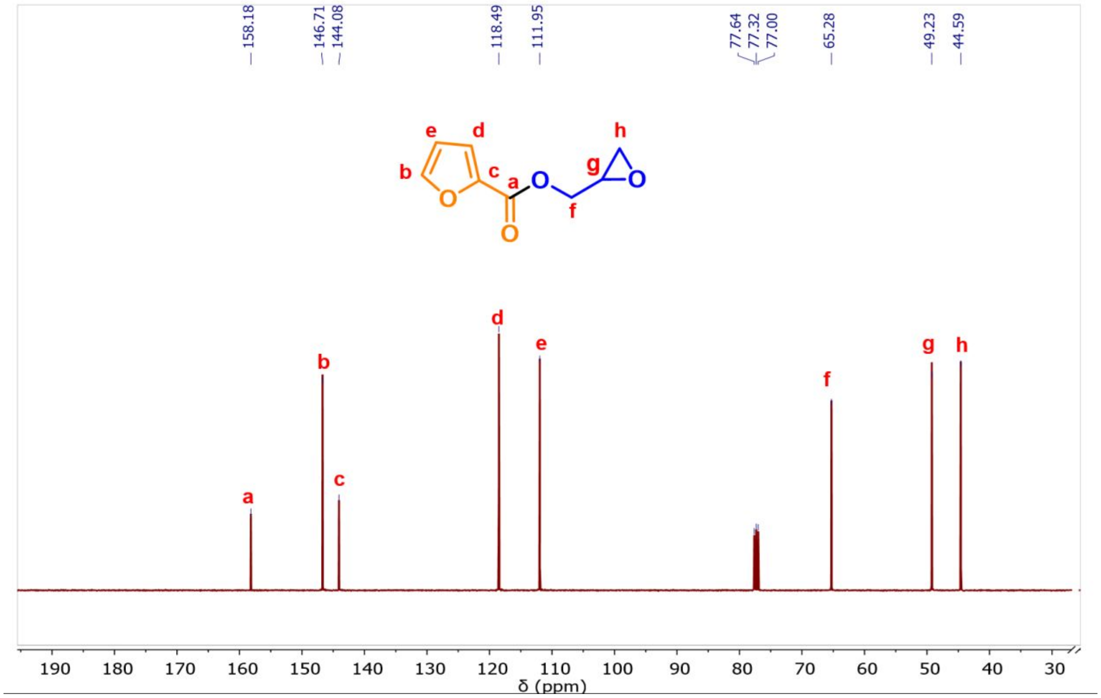

Figure S2: ^{13}C NMR spectrum (100 MHz, $CDCl_3$) of glycidyl furoate (GFu).

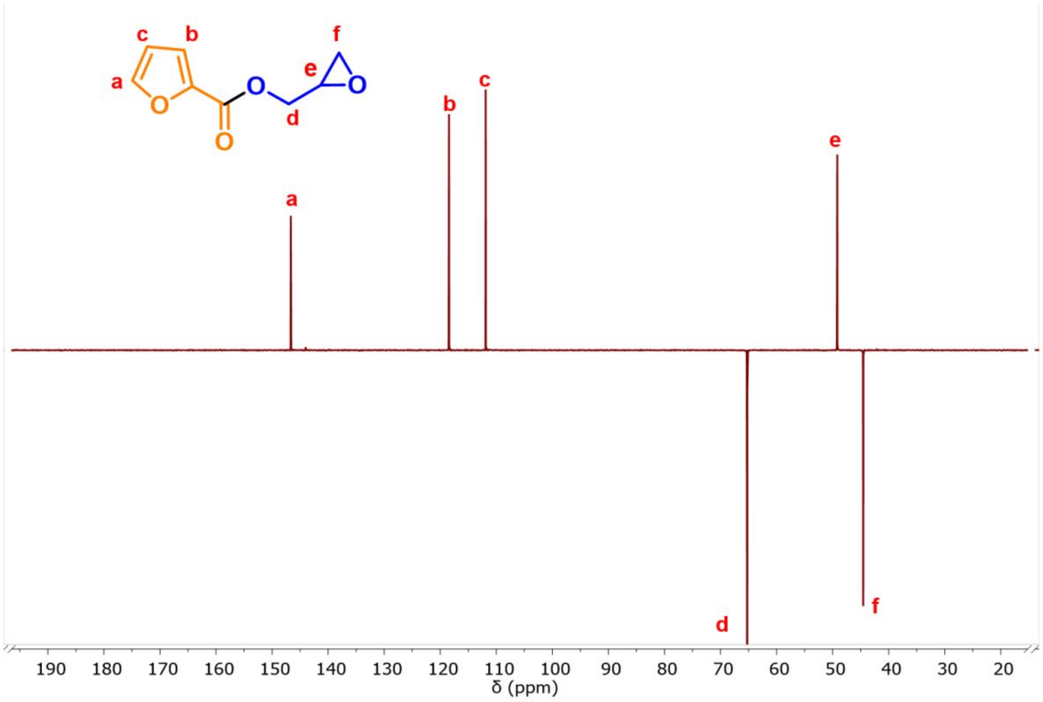

Figure S3: DEPT-135 spectrum of glycidyl furoate (GFu).

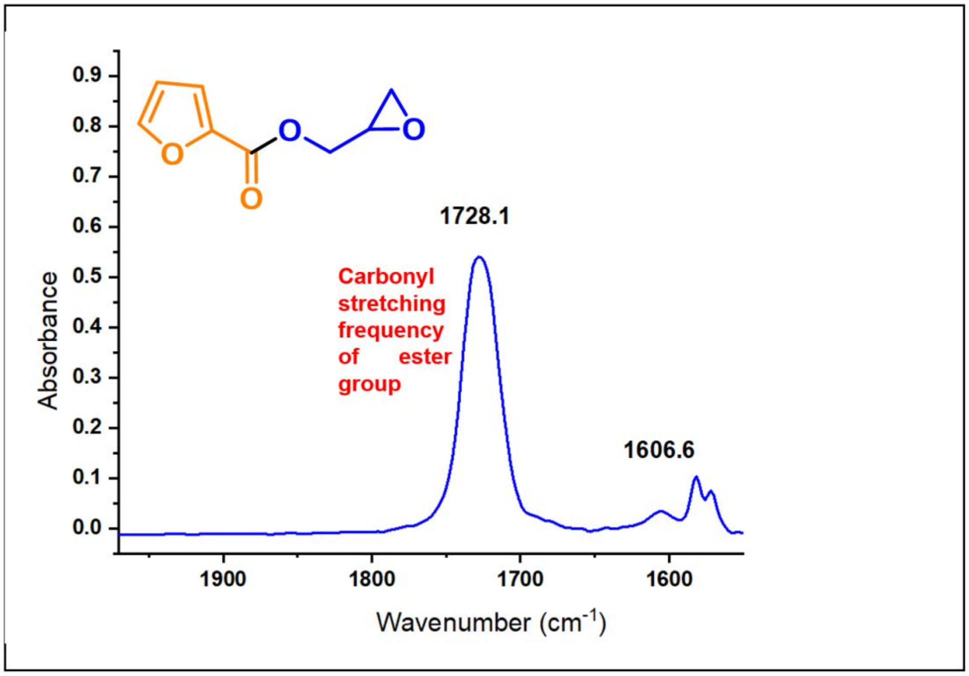

Figure S4: ATR FT-IR of glycidyl furoate (GFu) in CH_2Cl_2.

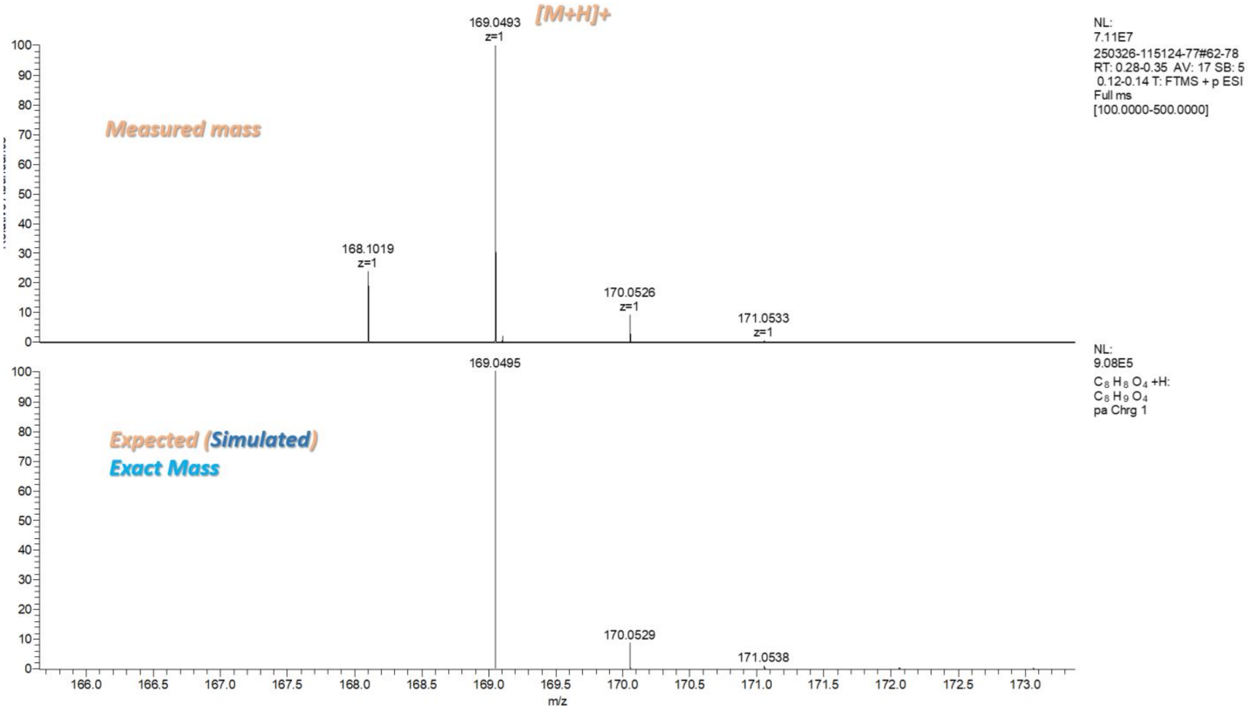

Figure S5: ESI-MS spectrum of glycidyl furoate (GFu).

Copolymerization of Glycidyl Furoate with CO_2.

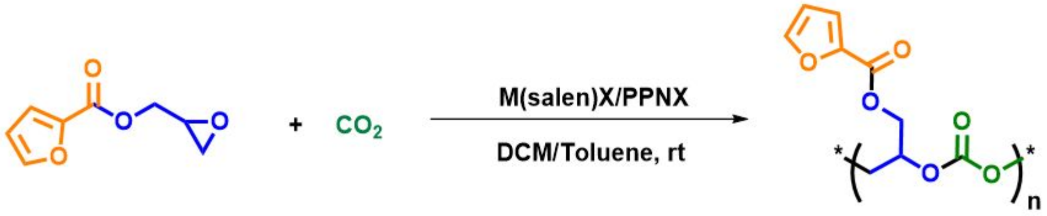

Scheme S3. Copolymerization of GFu with CO_2.

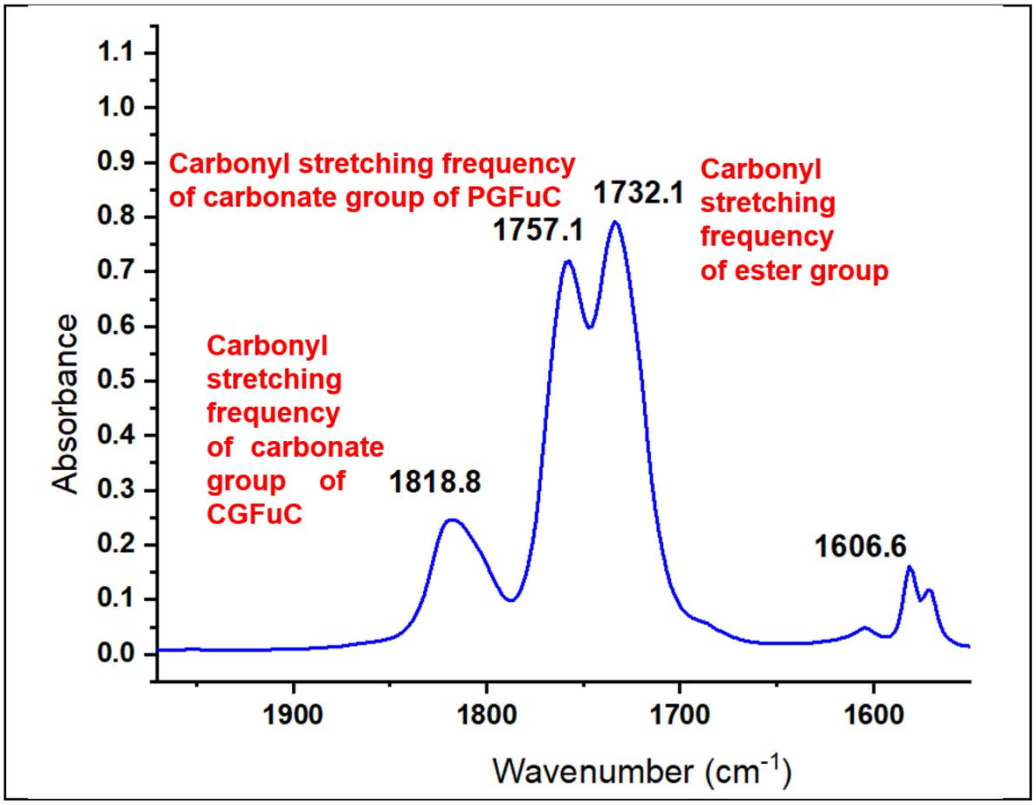

Figure S6: ATR FT-IR spectrum of reaction mixture of GFu with CO_2 after 24 h.

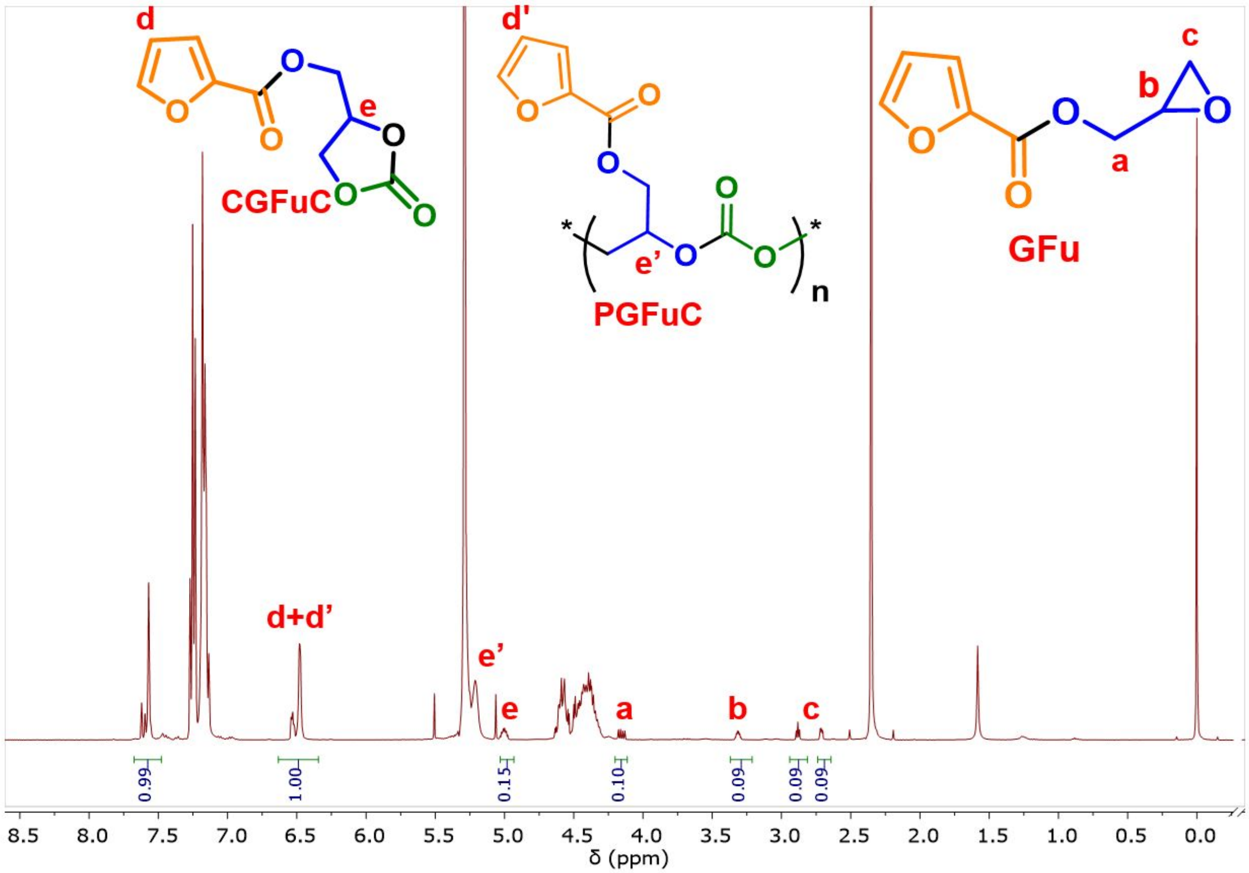

Figure S7: ^{1}H NMR spectrum (400 MHz, $CDCl_3$) of reaction mixture of GFu with CO_2 after 24 h.

The conversion of GFu monomer to PGFuC and CGFuC was determined by comparing the relative integral of **d+d’** (PGFuC+ CGFuC) and **c** (unreacted monomer).

The selectivity for the formation of PGFuC over CGFuC was determined by comparing the relative integral of **d+d’** (PGFuC+ CGFuC) and **e** (CGFuC).

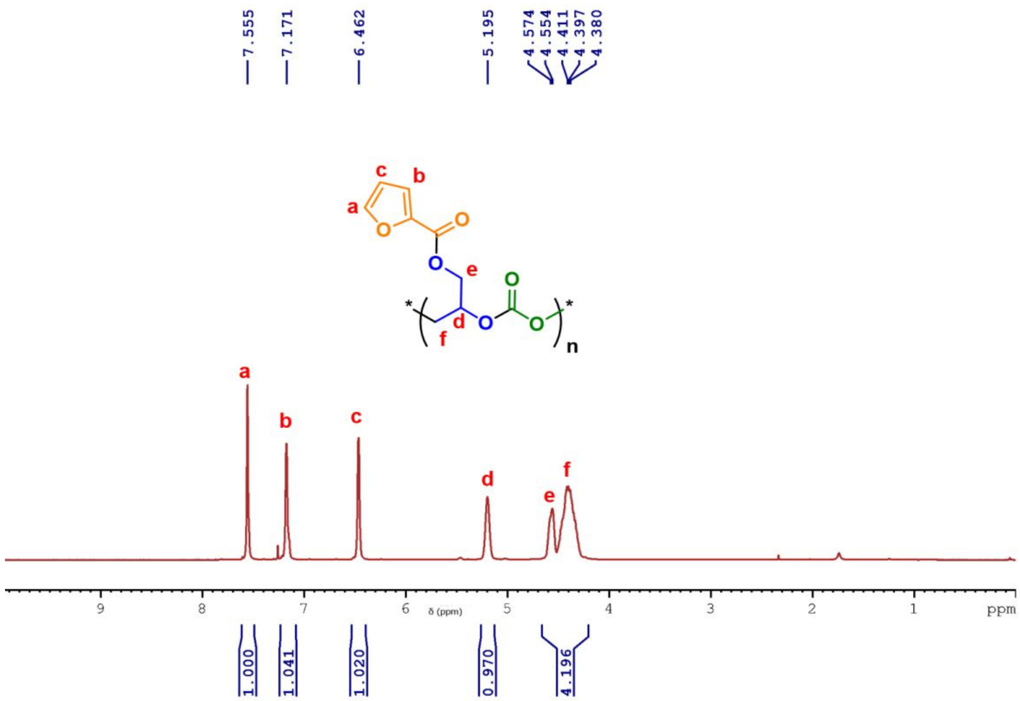

Figure S8: ^{1}H NMR spectrum (400 MHz, $CDCl_3$) of poly(glycidyl furoate) carbonate (PGFuC).

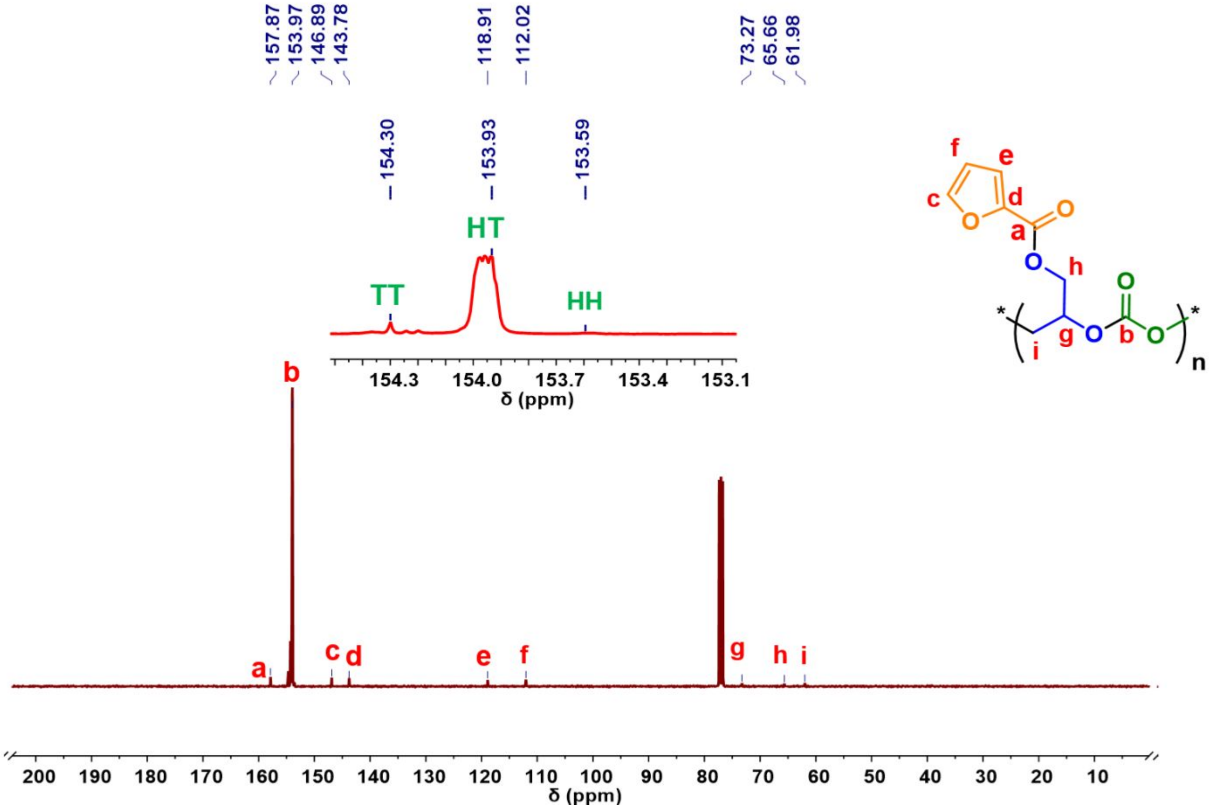

Figure S9: ^{13}C NMR spectrum (100 MHz, $CDCl_3$) of poly(glycidyl furoate) carbonate (PGFuC).

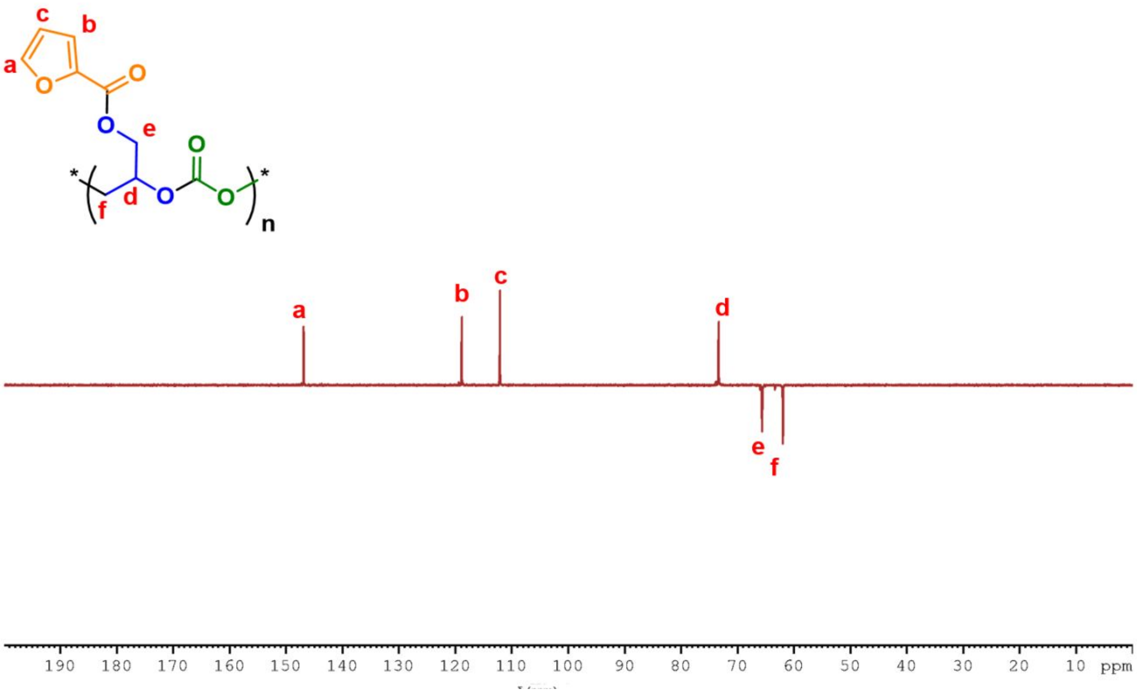

Figure S10: DEPT-135 spectrum of poly(glycidyl furoate) carbonate (PGFuC).

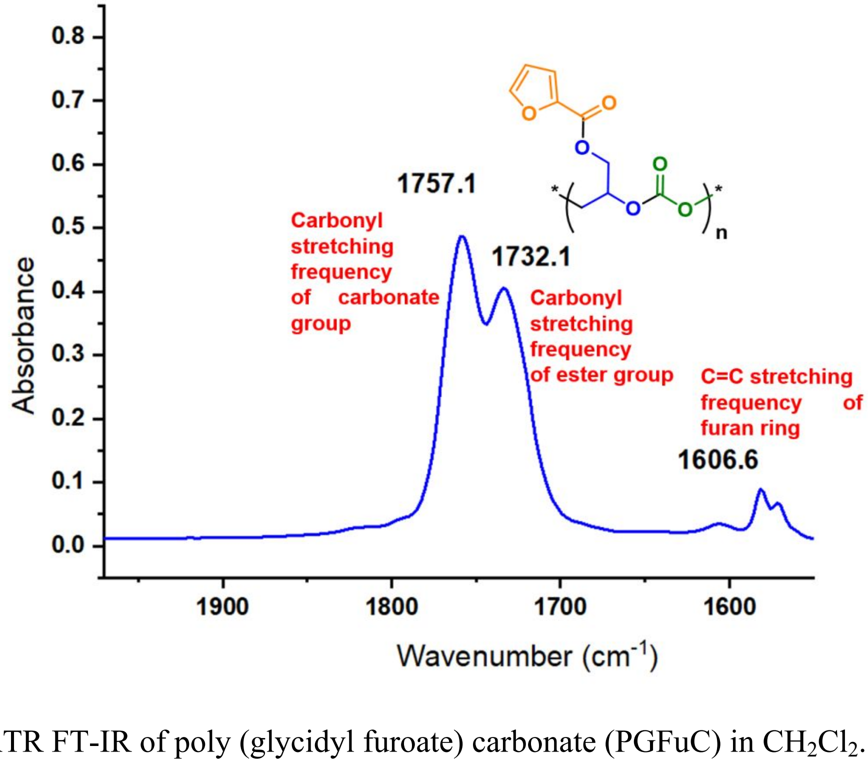

Figure S11: ATR FT-IR of poly (glycidyl furoate) carbonate (PGFuC) in CH_2Cl_2.

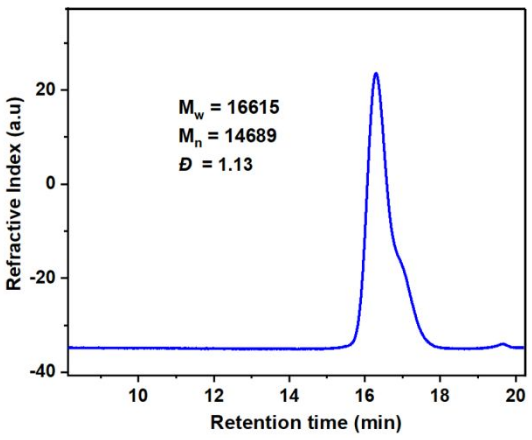

Figure S12: GPC trace of polycarbonate sample with catalyst **1** after 24 h (Table 1, Entry 1).

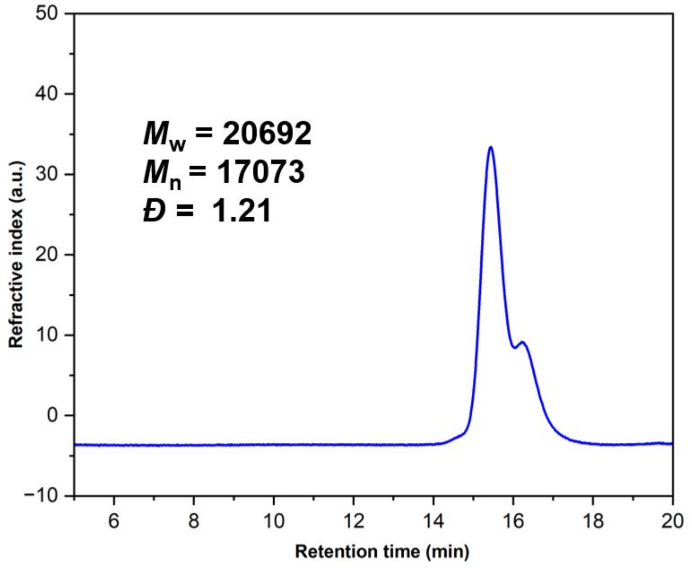

Figure S13: GPC trace of polycarbonate sample with catalyst **1** after 36 h (Table 1, Entry 2).

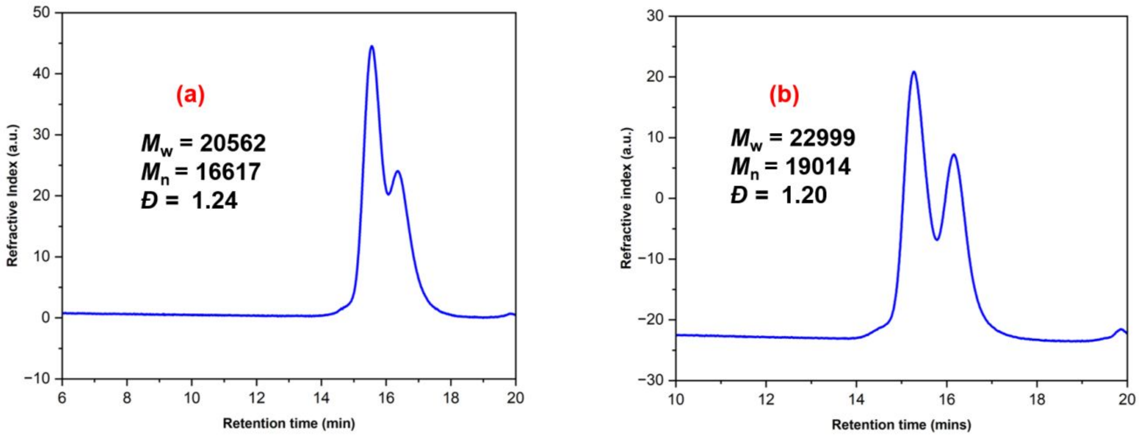

Figure S14: GPC trace of polycarbonate sample with (a) catalyst **2** (Table 1, Entry 4); (b) catalyst **3** (Table 1, Entry 5).

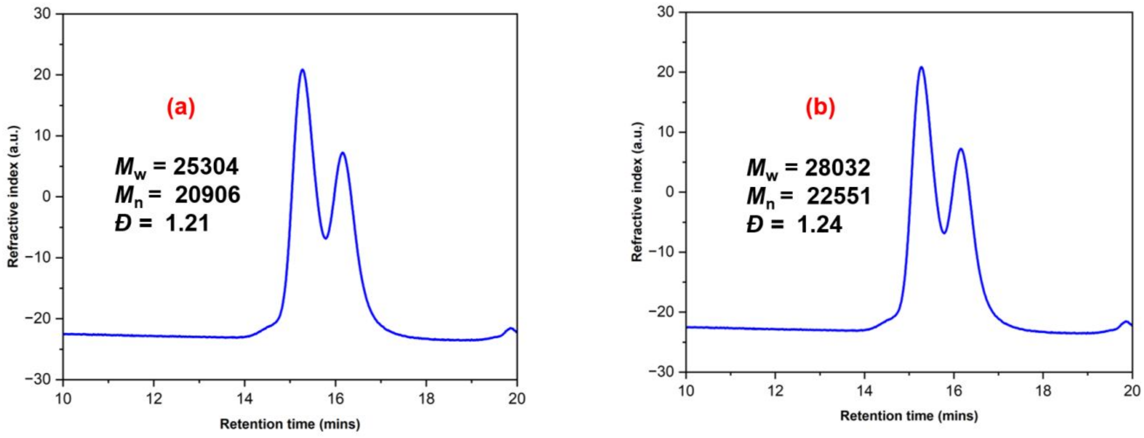

Figure S15: GPC trace of polycarbonate sample with catalyst **1** and different cocatalysts (a) $PPNN_3$ (Table 1, Entry 6); (b) PPNCl (Table 1, Entry 7).

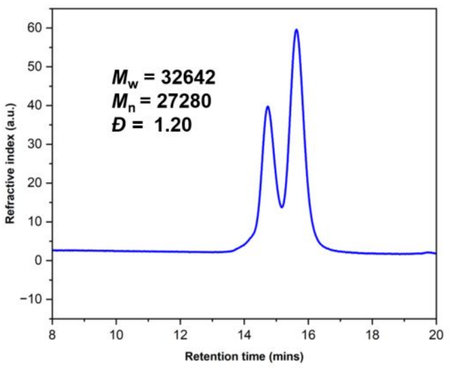

Figure S16: GPC trace of polycarbonate sample with increased loading of monomer (Table 1, Entry 8).

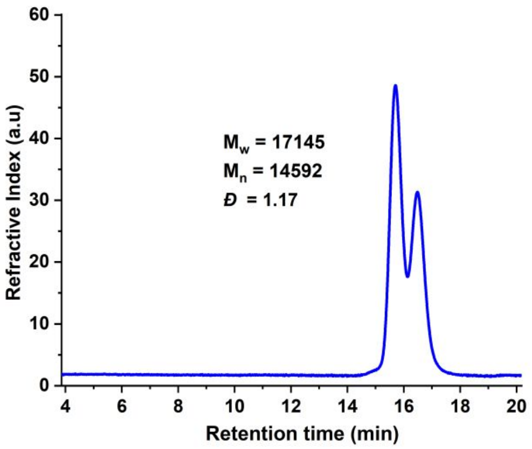

Figure S17: GPC trace of polycarbonate sample when reaction done in propylene carbonate as solvent (Table 1, Entry 9).

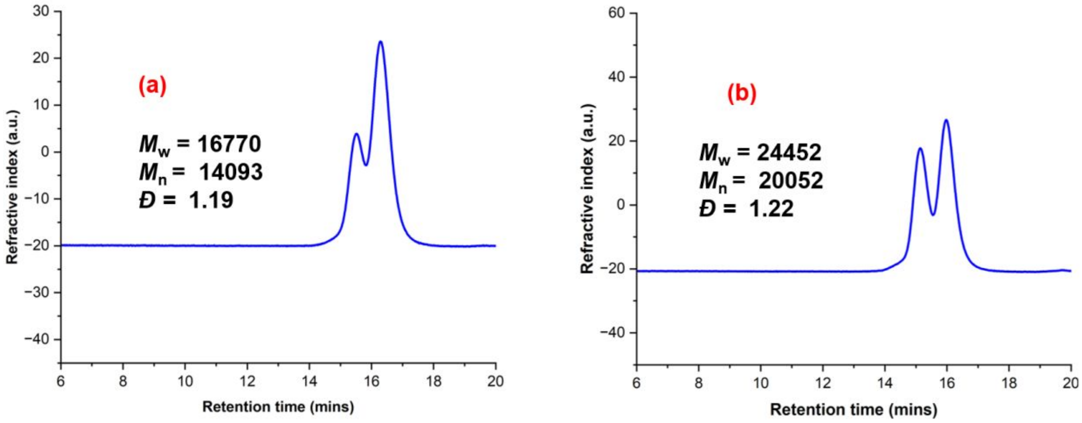

Figure S18: GPC trace of polycarbonate sample at varying CO_2 pressure (a) 5 bar (b) 15 bar.

Cyclic product formation

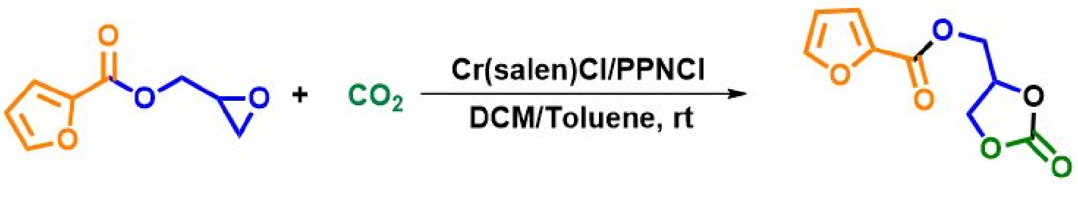

Scheme S4. Reaction of GFu with CO_2 to give cyclic carbonate.

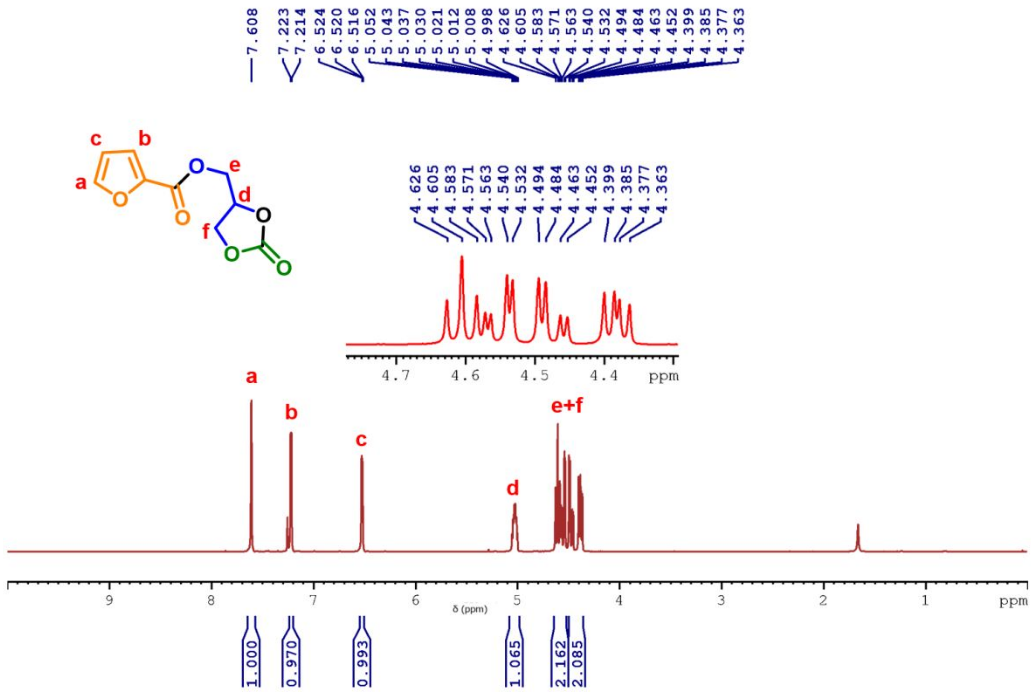

Figure S19: ^{1}H NMR spectrum (400 MHz, $CDCl_3$) of cyclic (glycidyl furoate) carbonate (CGFuC).

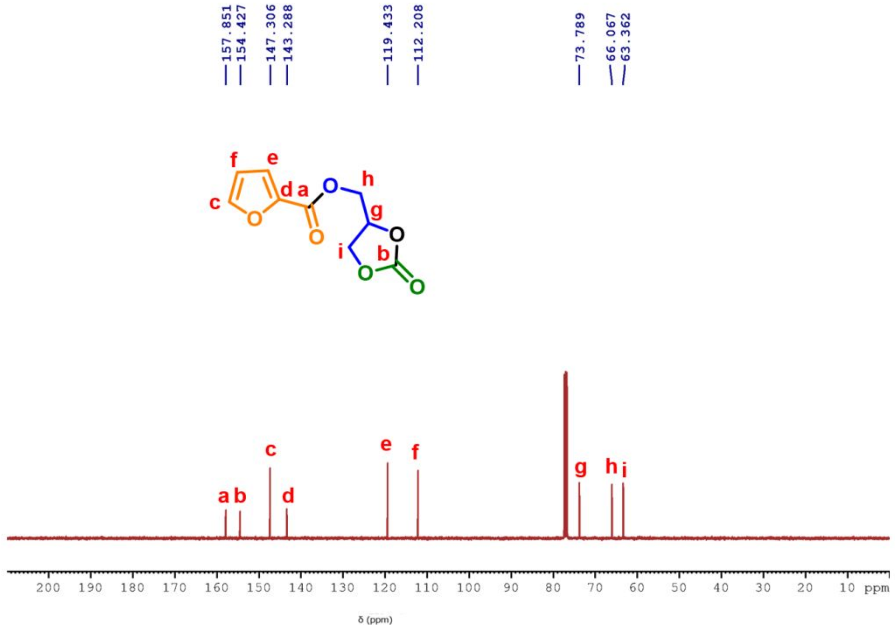

Figure S20: ^{13}C NMR spectrum (100 MHz, $CDCl_3$) of cyclic (glycidyl furoate) carbonate (CGFuC).

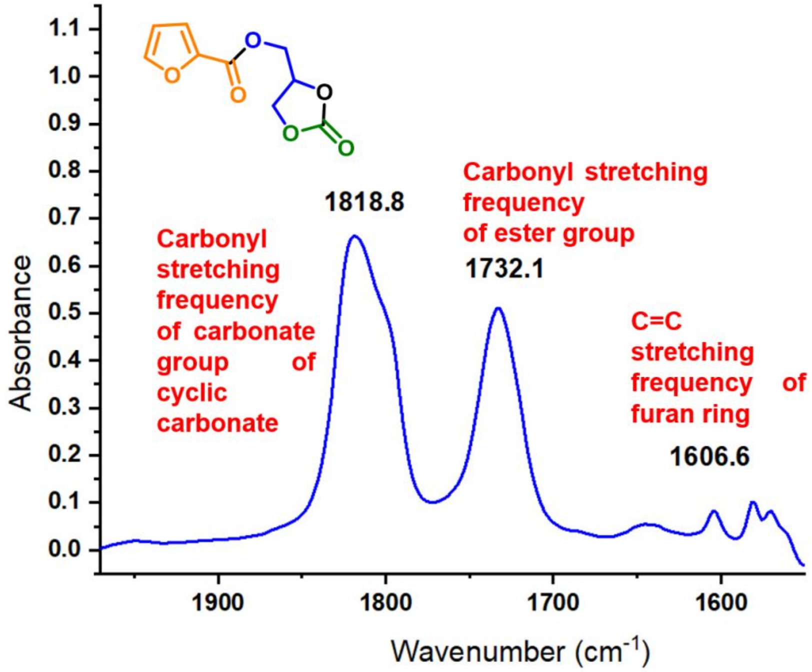

Figure S21: ATR FT-IR of cyclic (glycidyl furoate) carbonate (CGFuC) in CH_2Cl_2.

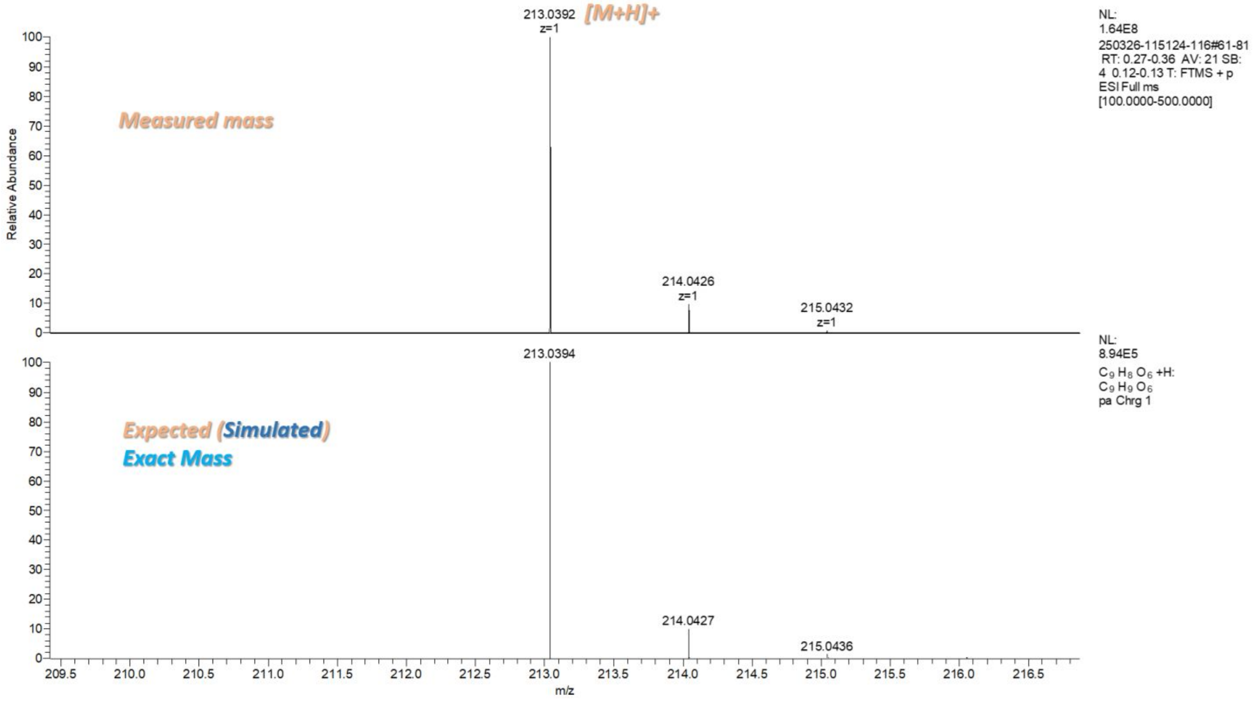

Figure S22: ESI-MS spectrum of cyclic (glycidyl furoate)carbonate (CGFuC).

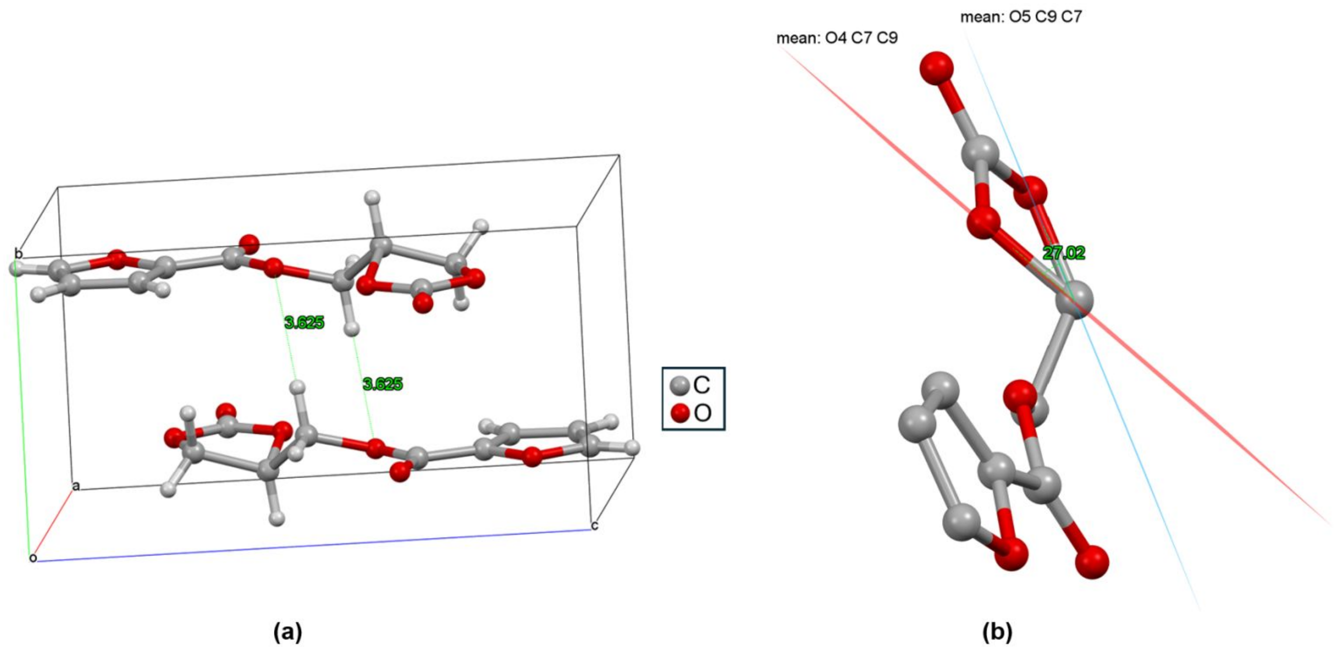

Figure S23: (a) Molecular packing structure of cyclic (glycidyl furoate)carbonate (CGFuC). **(b)** Dihedral angle between the planes of O4 C7 C9 and O5 C9 C7.

Table S1. Crystal data and data collection parameters for CGFuC (CCDC 2477314).

Empirical formula	$C_9H_8O_6$	
Formula weight	212.15	
Temperature	100 K	
Crystal system	Triclinic	
Space group	*P*-1	
Unit cell dimensions	*a* = 6.2821(9) Å	α = 89.478(7)°
	b = 6.3397(9) Å	β = 81.436(5)°
	c = 11.4568(16) Å	γ = 89.936(6)°
Volume	451.18(11) Å^3	
Z	2	
Radiation	Cu Kα (λ = 1.54178 Å)	
Density (calculated)	1.562 g cm^{-3}	
Absorption coefficient (*m*)	1.170 mm^{-1}	
F(000)	220	
Crystal size	0.206 x 0.201 x 0.12 mm^3	
Theta range for data collection	3.902 to 74.854°	
Index ranges	-7<=h<=7, -7<=k<=7, -14<=l<=14	
Reflections collected	15474	
Independent reflections	1827 [R(int) = 0.0567]	
Completeness to theta = 67.679 °	99.1 %	
Refinement method	Full-matrix least-squares on F^2	
Data/restraints/parameters	1827 / 0 / 156	
Goodness-of-fit on F^2 (GooF)	1.162	
Final R indices [I>2σ(I)]	R_1 = 0.0920, wR_2 = 0.2638	
R indices (all data)	R_1 = 0.0952, wR_2 = 0.2666	
Largest diff. peak and hole	0.509 and -0.334 e. Å^{-3}	

[a]$R_1=\Sigma(||F_o|-|F_c||)/\Sigma|F_o|$. [b]$wR_2=[\Sigma[w(F_o^2-F_c^2)^2]/\Sigma[w(F_o^2)^2]]^{1/2}$, $w=1/[\sigma^2(F_o^2)+(ap)^2+bp]$, where $p=[\max(F_o^2, 0)+2F_c^2]/3$.

Table S2. Bond distance parameters for CGFuC.

**Bond length (Å)**	**CGFuC**
O1-C1	1.369(6)
O1-C4	1.364(5)
O2-C5	1.205(5)
O3-C5	1.352(5)
O3-C6	1.466(12)
O3-C6A	1.464(10)
O4-C7	1.498(7)
O4-C7A	1.493(7)
O4-C8	1.331(5)
O5-C8	1.335(4)
O5-C9	1.454(4)
O6-C8	1.190(5)
C1-C2	1.340(7)
C2-C3	1.419(6)
C3-C4	1.344(6)
C4-C5	1.450(6)
C7-C9	1.566(9)
C7-C6	1.53(2)
C7A-C9	1.563(9)
C7A-C6A	1.45(2)

Table S3. Bond angle parameters for CGFuC.

Bond angle (°)	CGFuC
C4-O1-C1	105.4(4)
C5-O3-C6	116.0(5)
C5-O3-C6A	117.0(4)
C8-O4-C7	107.9(4)
C8-O4-C7A	108.4(4)
C8-O5-C9	109.7(3)
C2-C1-O1	111.1(4)
C1-C2-C3	106.1(4)
C4-C3-C2	106.6(4)
O1-C4-C5	116.5(4)
C3-C4-O1	110.8(4)
C3-C4-C5	132.7(4)
O2-C5-O3	124.3(4)
O2-C5-C4	126.6(4)
O3-C5-C4	109.1(3)
O4-C7-C9	99.1(5)
O4-C7-C6	104.2(9)
C6-C7-C9	108.1(9)
O4-C7A-C9	99.4(5)
C6A-C7A-O4	106.8(10)
C6A-C7A-C9	110.3(9)
O4-C8-O5	112.2(3)
O6-C8-O4	123.8(4)
O6-C8-O5	124.0(4)
O5-C9-C7	102.1(4)
O5-C9-C7A	102.4(4)
O3-C6-C7	103.5(12)
C7A-C6A-O3	106.7(13)

Copolymerization of GFu with COS:

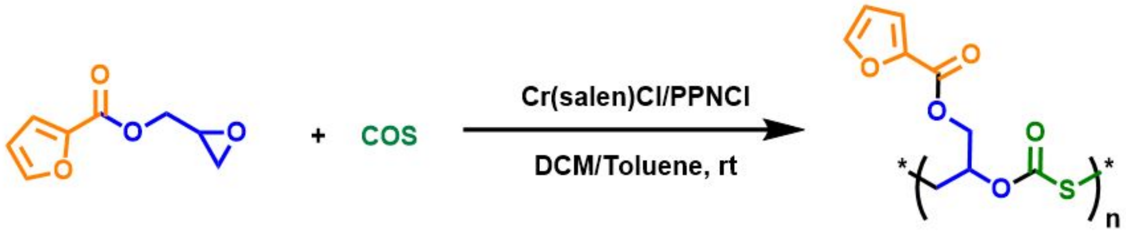

Scheme S5. Copolymerization of GFu with COS.

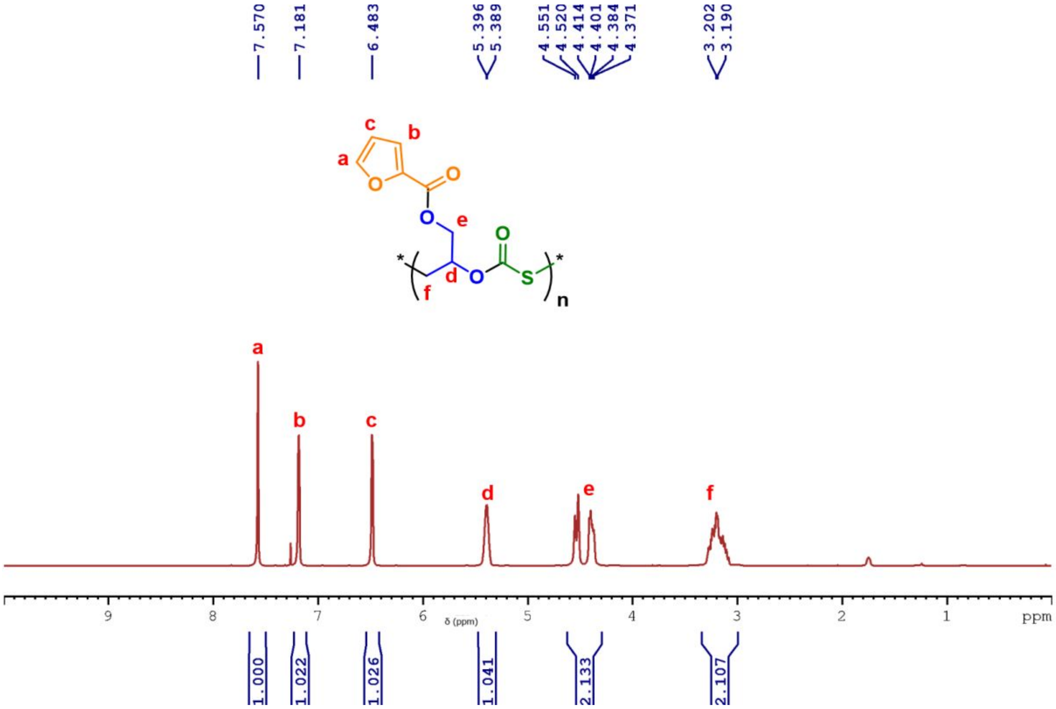

Figure S24: ^{1}H NMR spectrum (400 MHz, $CDCl_3$) of poly(glycidyl furoate) monothioocarbonate (PGFuMTC).

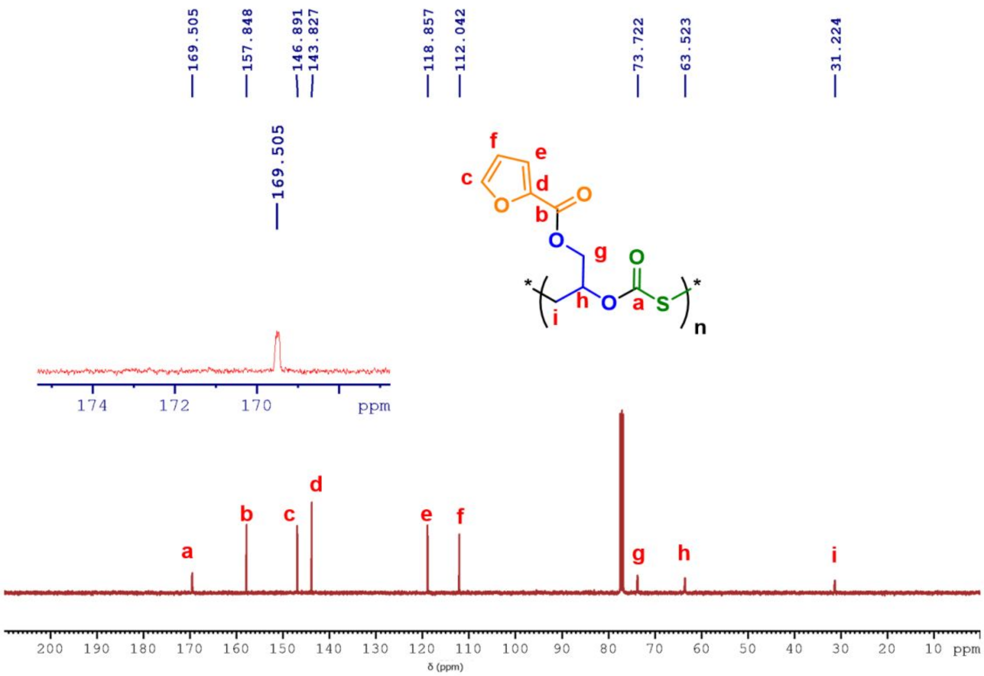

Figure S25: ^{13}C NMR spectrum (100 MHz, $CDCl_3$) of poly(glycidyl furoate) monothiocarbonate (PGFuMTC).

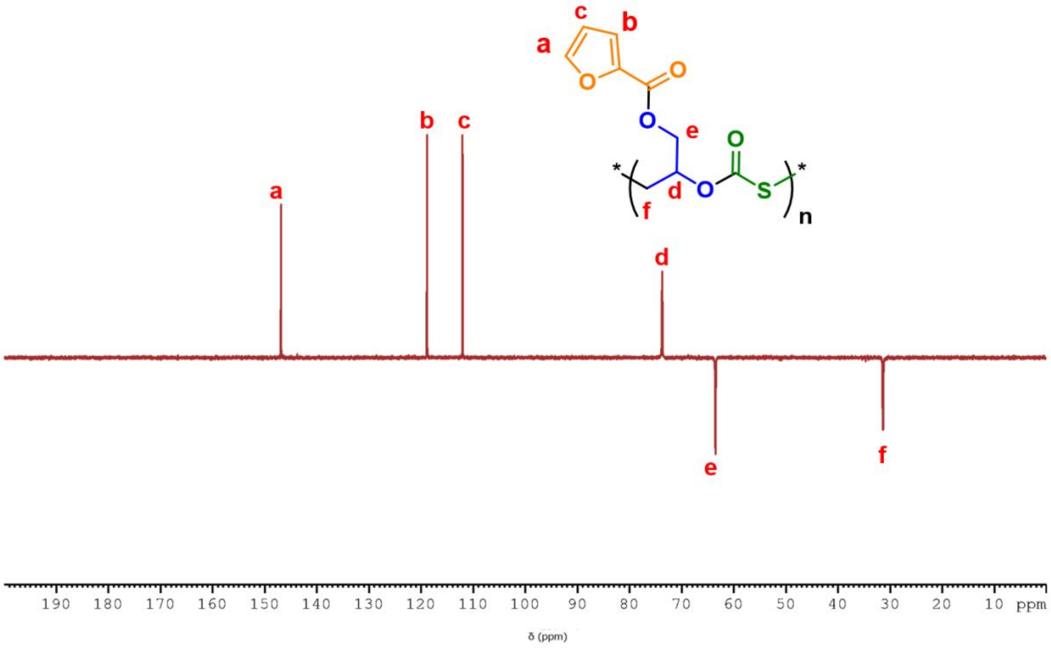

Figure S26: DEPT-135 spectrum of poly(glycidyl furoate) monothiocarbonate (PGFuMTC).

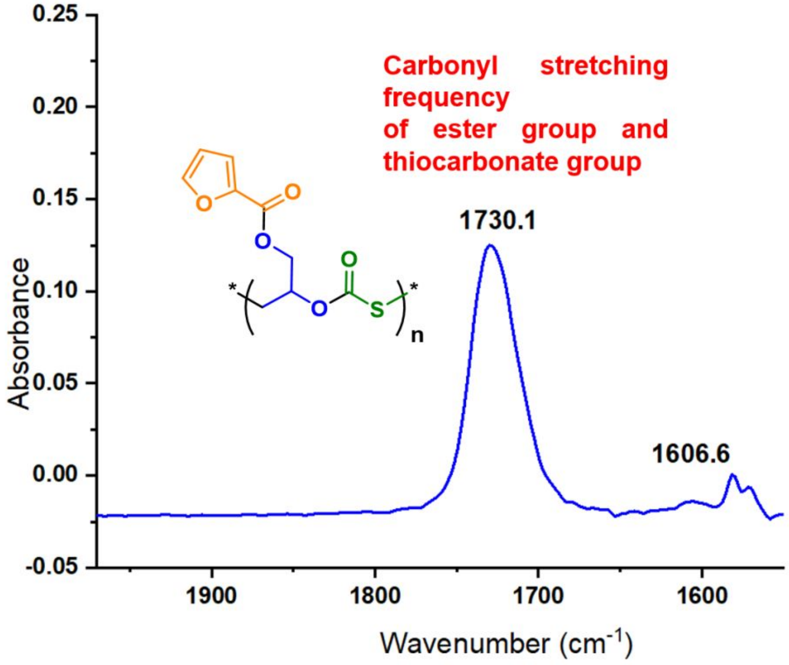

Figure S27: ATR FT-IR of poly(glycidyl furoate) monothiocarbonate (PGFuMTC) in CH_2Cl_2.

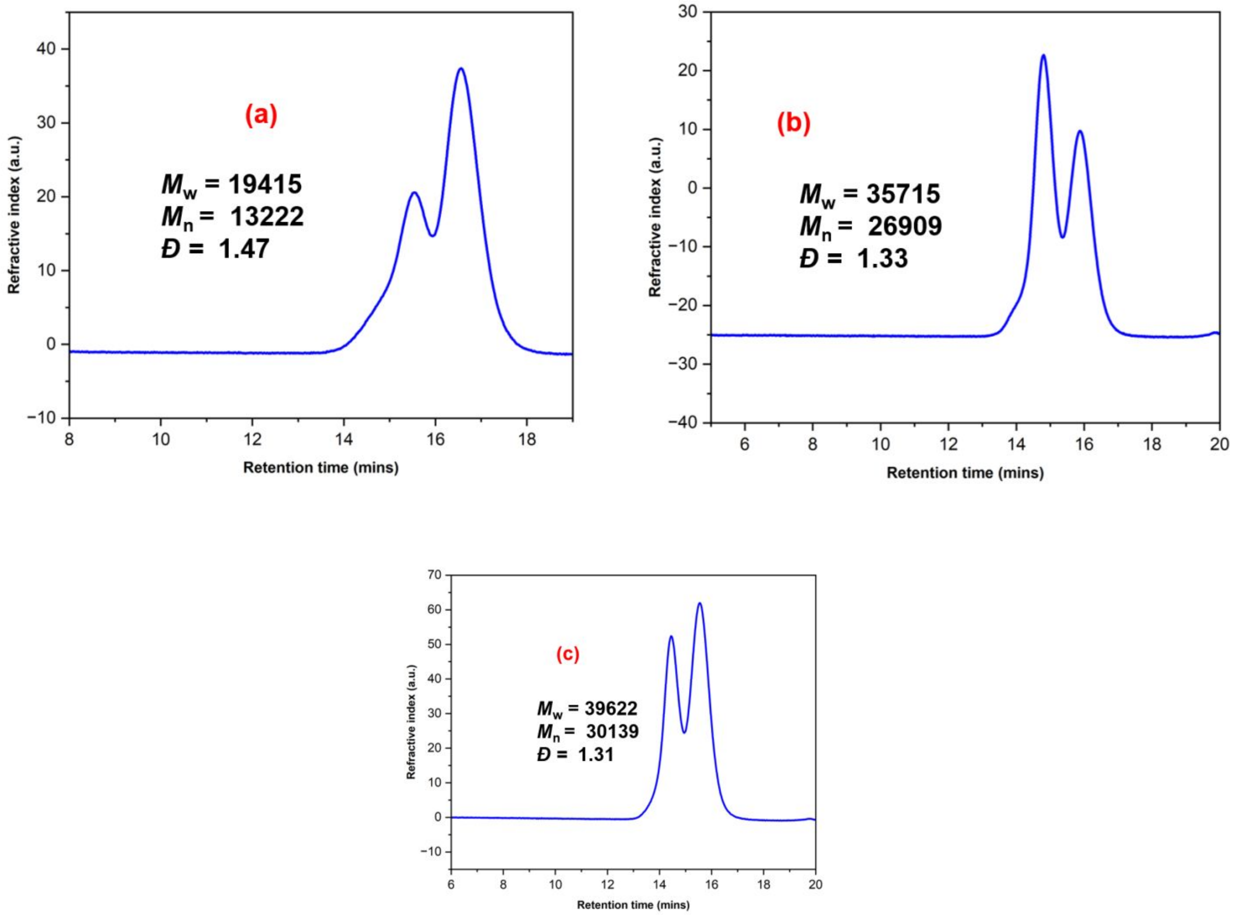

Figure S28: GPC trace of poly(monothiocarbonate) sample at three different monomer loading (a) 250 equivalents (b) 500 equivalents (c) 750 equivalents.

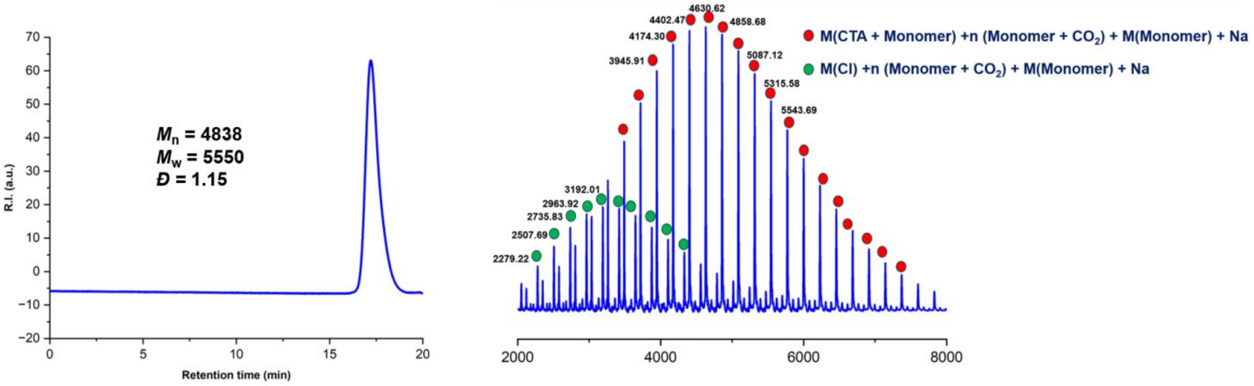

Figure 29 (a) GPC trace of oligomer of GFu/COS copolymer. **(b)** MALDI-TOF spectrum of GFu/COS copolymer.

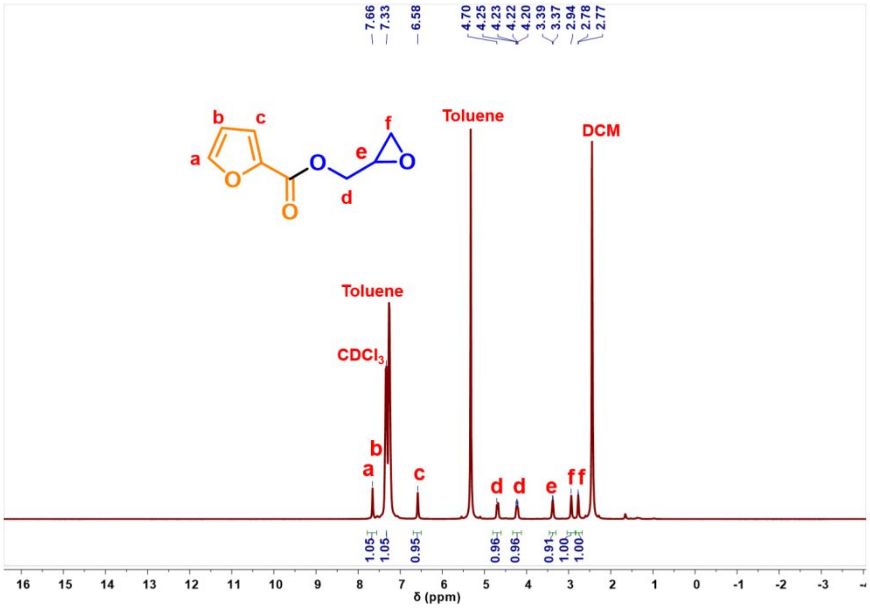

Figure S30: ^{1}H NMR spectrum (400 MHz, $CDCl_3$) of reaction mixture of terpolymerization of GFu, COS and CO_2 using Co(III)TFA/PPNTFA at ambient temperature and 1.0 MPa CO_2 and 1.0 MPa COS pressure.

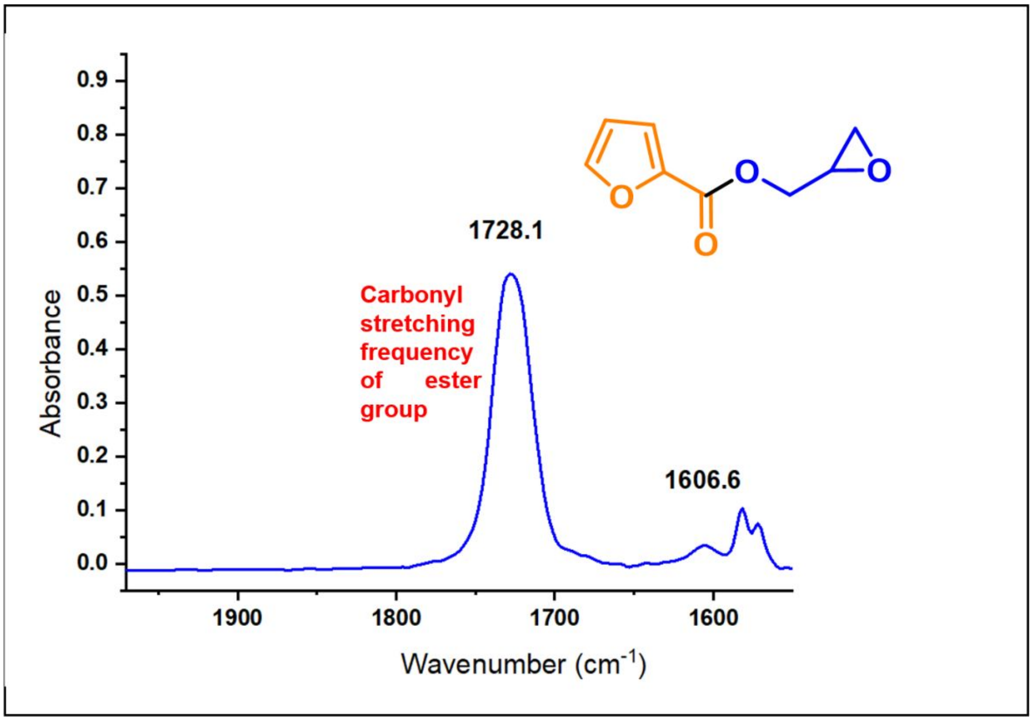

Figure S31: ATR FT-IR spectrum of reaction mixture of terpolymerization of GFu, COS and CO_2 using Co(III)TFA/PPNTFA at ambient temperature and 1.0 MPa CO_2 and 1.0 MPa COS pressure.

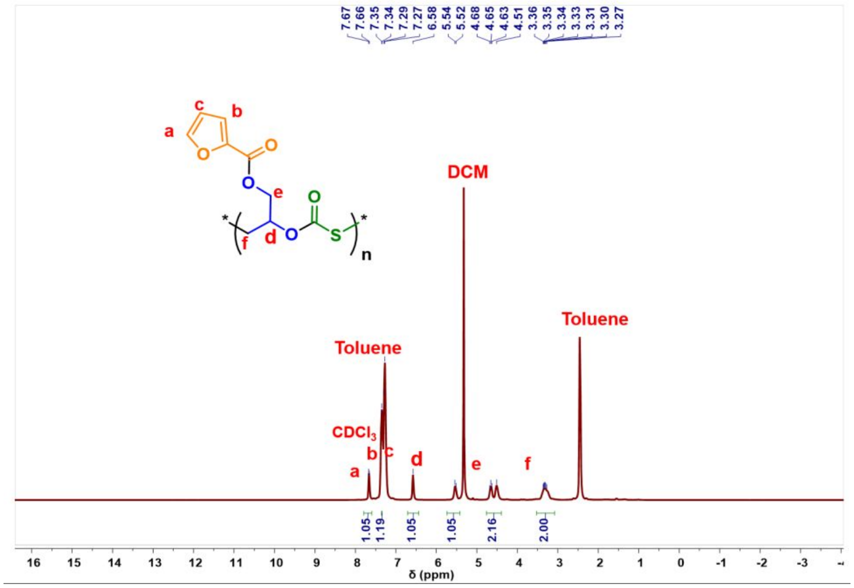

Figure S32: ^{1}H NMR spectrum (400 MHz, $CDCl_3$) of reaction mixture of terpolymerization of GFu, COS and CO_2 using Cr(III)Cl/PPNCl at ambient temperature and 1.0 MPa CO_2 and 1.0 MPa COS pressure.

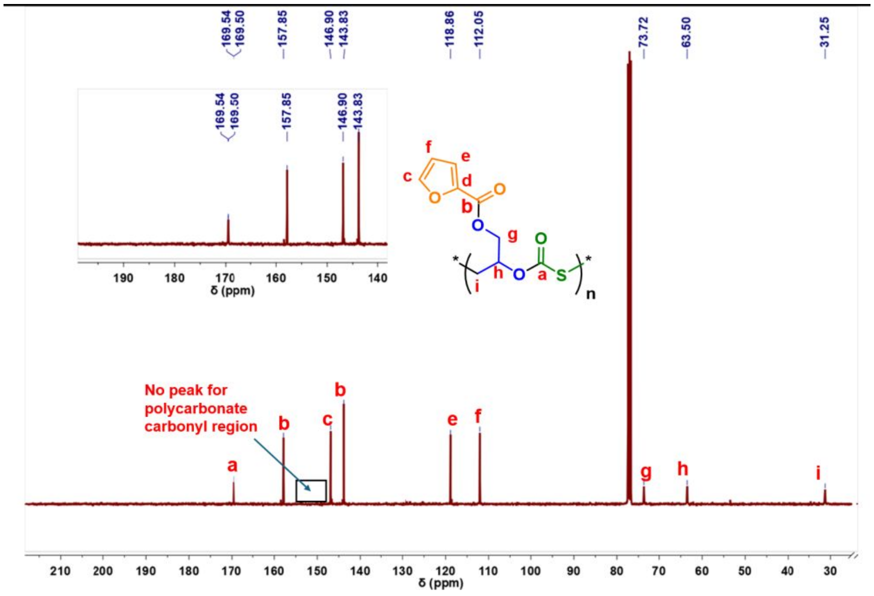

Figure S33: ^{13}C NMR spectrum (400 MHz, $CDCl_3$) of reaction mixture of terpolymerization of GFu, COS and CO_2 using Cr(III)Cl/PPNCl at ambient temperature and 1.0 MPa CO_2 and 1.0 MPa COS pressure.

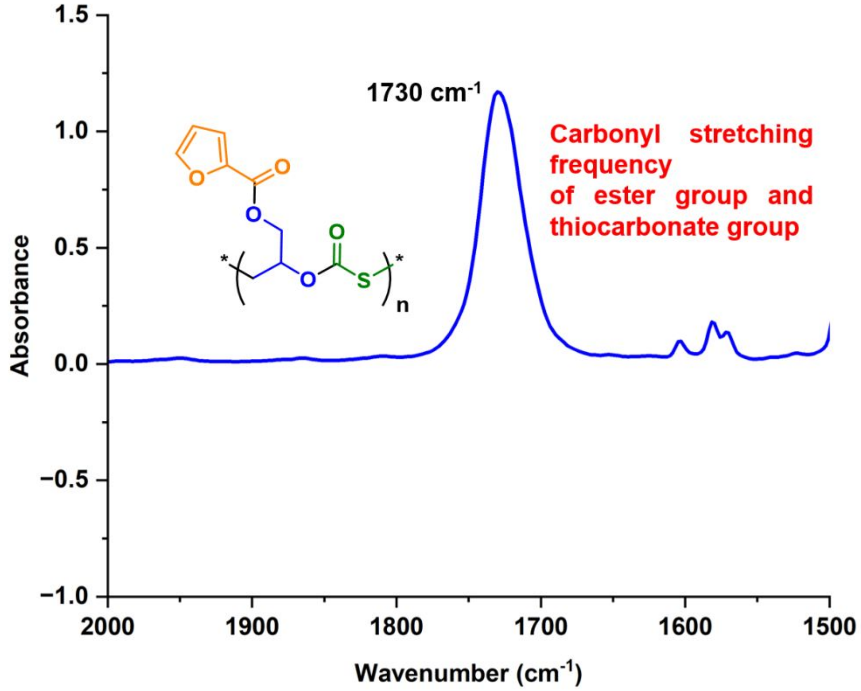

Figure S34: ATR FT-IR spectrum of reaction mixture of terpolymerization of GFu, COS and CO_2 using Cr(III)Cl/PPNCl at ambient temperature and 1.0 MPa CO_2 and 1.0 MPa COS pressure.

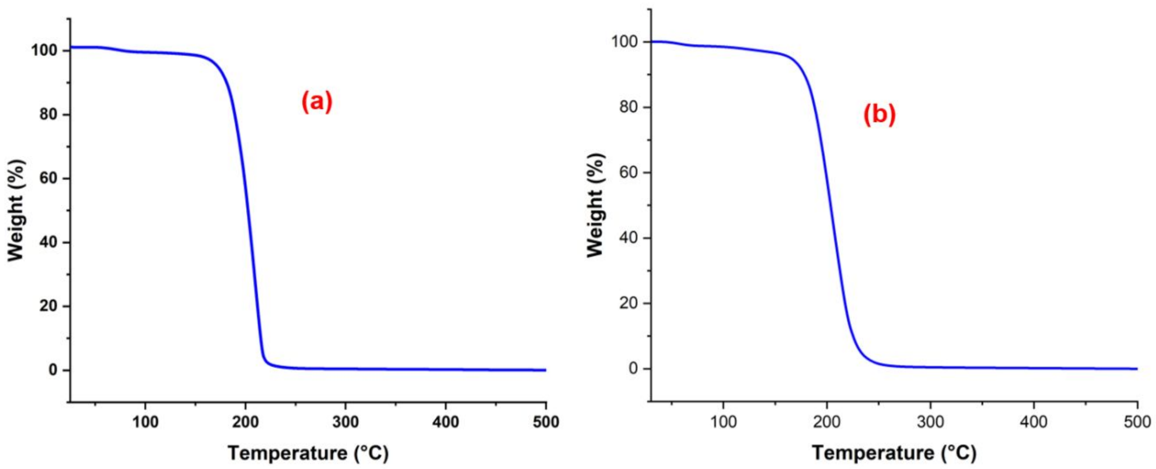

Figure S35 TGA trace for (a) Polycarbonate (b) Poly(monothiocarbonate).

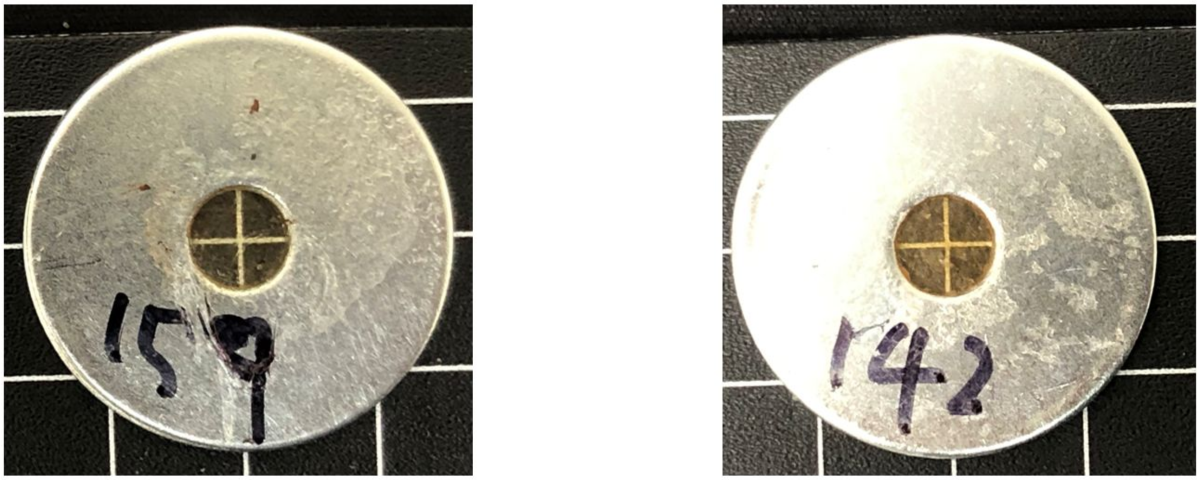

(a) Polycarbonate (b) Poly(monothiocarbonate)

Figure S36 Samples prepared for nanoindentation test.

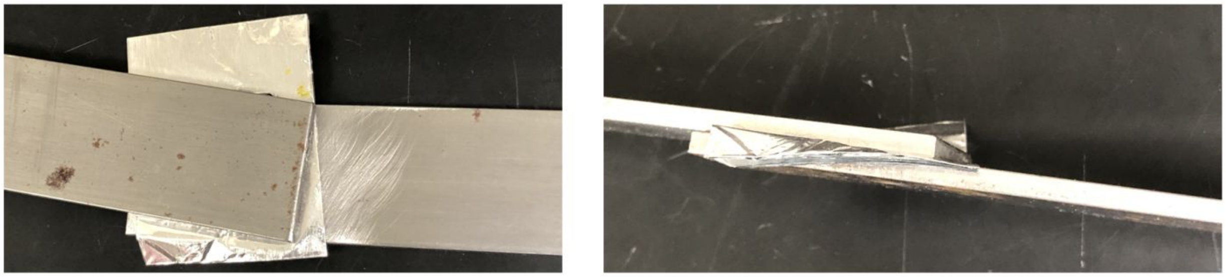

(a) Polycarbonate (b) Poly(monothiocarbonate)

Figure S37 Samples prepared for lap shear test.

Procedure for Polycarbonate Degradation

The polycarbonate sample (200 mg, 0.94 mmol) was dissolved in THF (2 mL). The solution was added to 1.0 (N) aq. NaOH (2 mL) and stirred for 60 minutes at an ambient temperature. The reaction was stopped, and the aqueous phase was extracted with dichloromethane (2 x 20 mL). The combined organic phases dried over anhydrous Na_2SO_4 and evaporation of the solvent by using rotary evaporation gave a colorless oily liquid (154 mg, Yield = 88%).

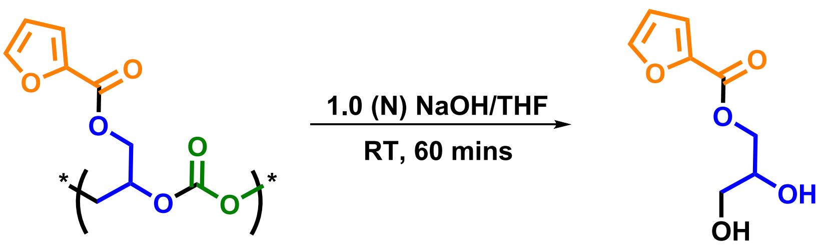

Scheme S6 Polycarbonate hydrolysis to diol.

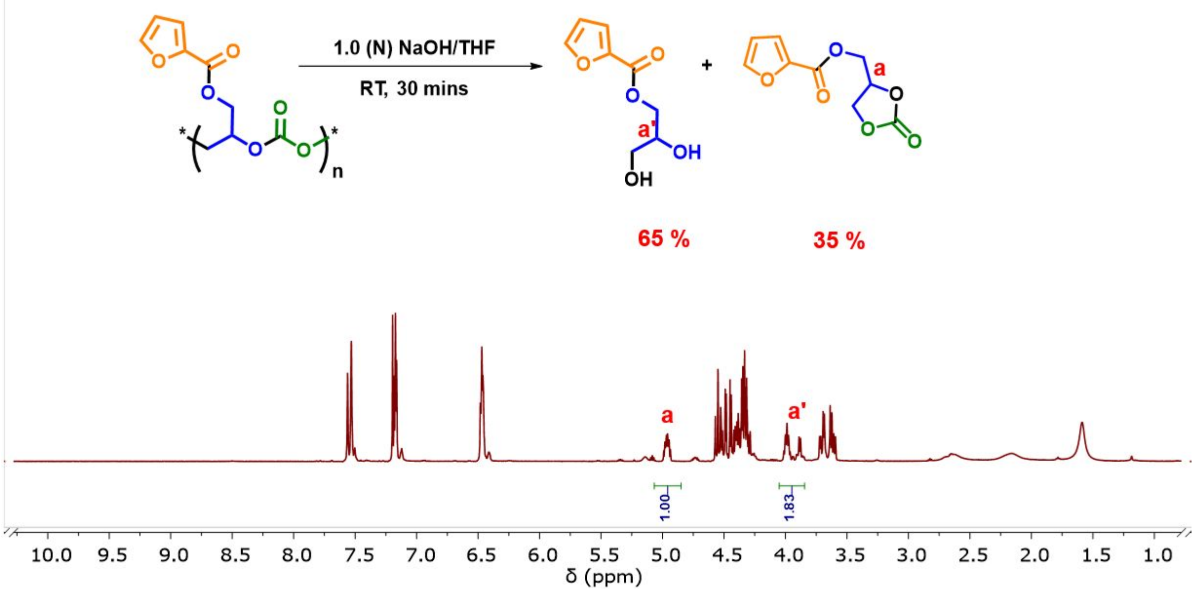

Figure S38 ^{1}H NMR spectrum (400 MHz, $CDCl_3$) of hydrolysis of poly(glycidyl furoate) carbonate (PGFuC) with aq.1.0 (N) NaOH after 30 minutes.

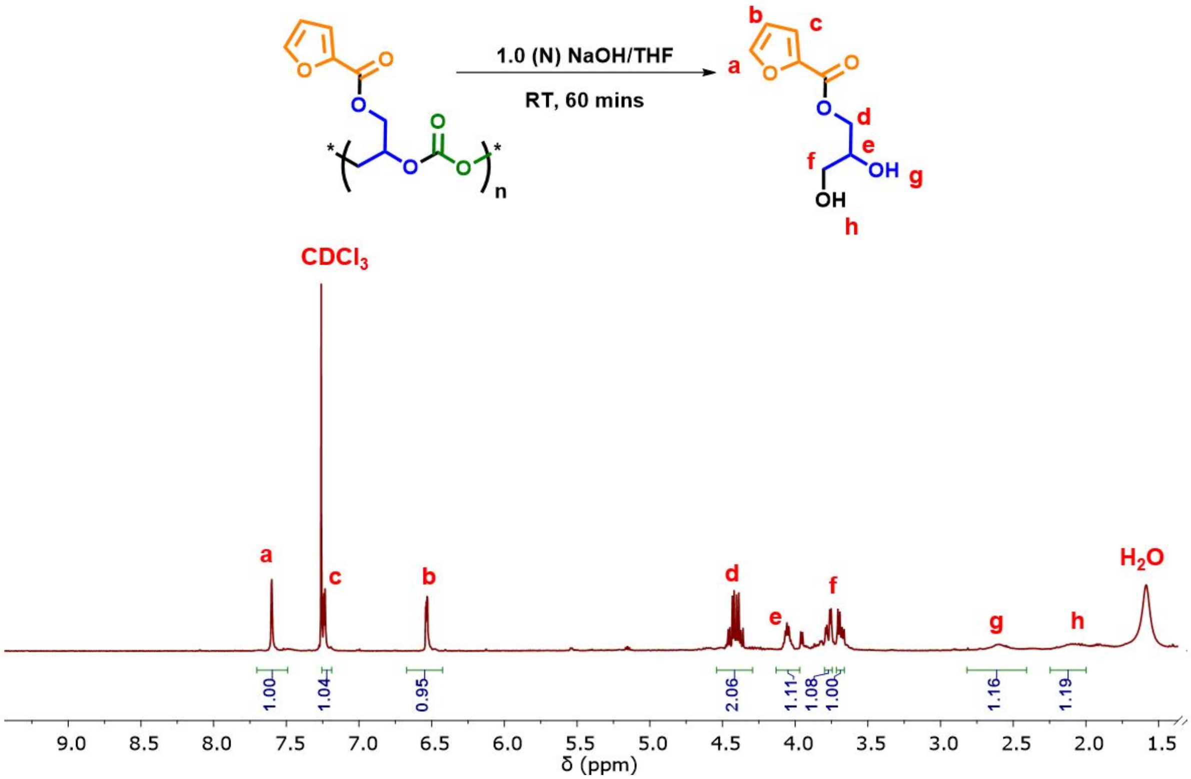

Figure S39 ^{1}H NMR spectrum (400 MHz, $CDCl_3$) of hydrolysis of poly(glycidyl furoate) carbonate (PGFuC) with aq.1.0 (N) NaOH after 60 minutes.

Procedure for preparation of glycidyl furoate epoxide (GFu) from diol

To a solution of diol (0.6 mmol, 112 mg, 1 equiv) in CH_2Cl_2 (20 mL) were added *p*-toluenesulfonyl chloride (0.72 mmol, 137 mg, 1.2 equiv), DMAP (0.06 mmol, 7.3 mg, 0.1 equiv), and triethylamine (1.2 mmol, 0.16 mL, 2 equiv) under constant stirring at room temperature. The reaction mixture was stirred for 4 h and then washed with water, dried over anhydrous Na_2SO_4. The solvent was removed on a rotary evaporator to give a residue that was purified on a silica gel column chromatography using hexane and ethyl acetate as an eluent to give tosylate as a colorless oil (Yield = 82%, 135 mg).

To a stirred solution of tosylate (0.36 mmol, 100 mg, 1 equiv) in methanol (10 mL) was added anhydrous K_2CO_3 (0.72 mmol, 100 mg, 2 equiv) at 0 °C. The mixture was then warmed and stirred at 25 °C for 1 h. The reaction mixture was concentrated, and ethyl acetate (20 mL) was added, then washed with water (20 mL). Organic layer dried over anhydrous Na_2SO_4 and evaporation of

the solvent gave a residue that was purified on a silica gel column chromatography using hexane and ethyl acetate as an eluent afford epoxide with 85% yield (51 mg).

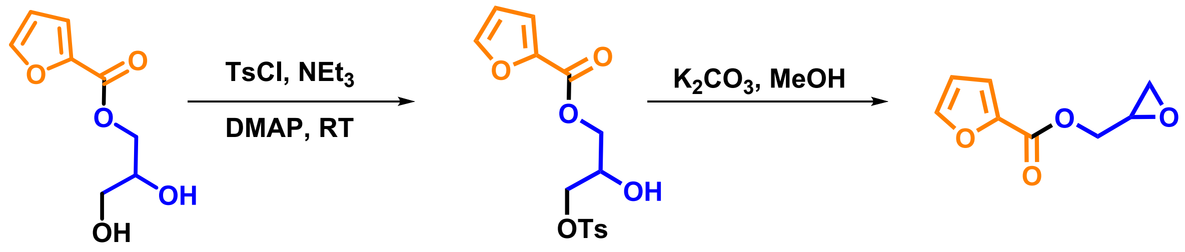

Scheme S7 Synthesis of glycidyl furoate epoxide (GFu) from diol.

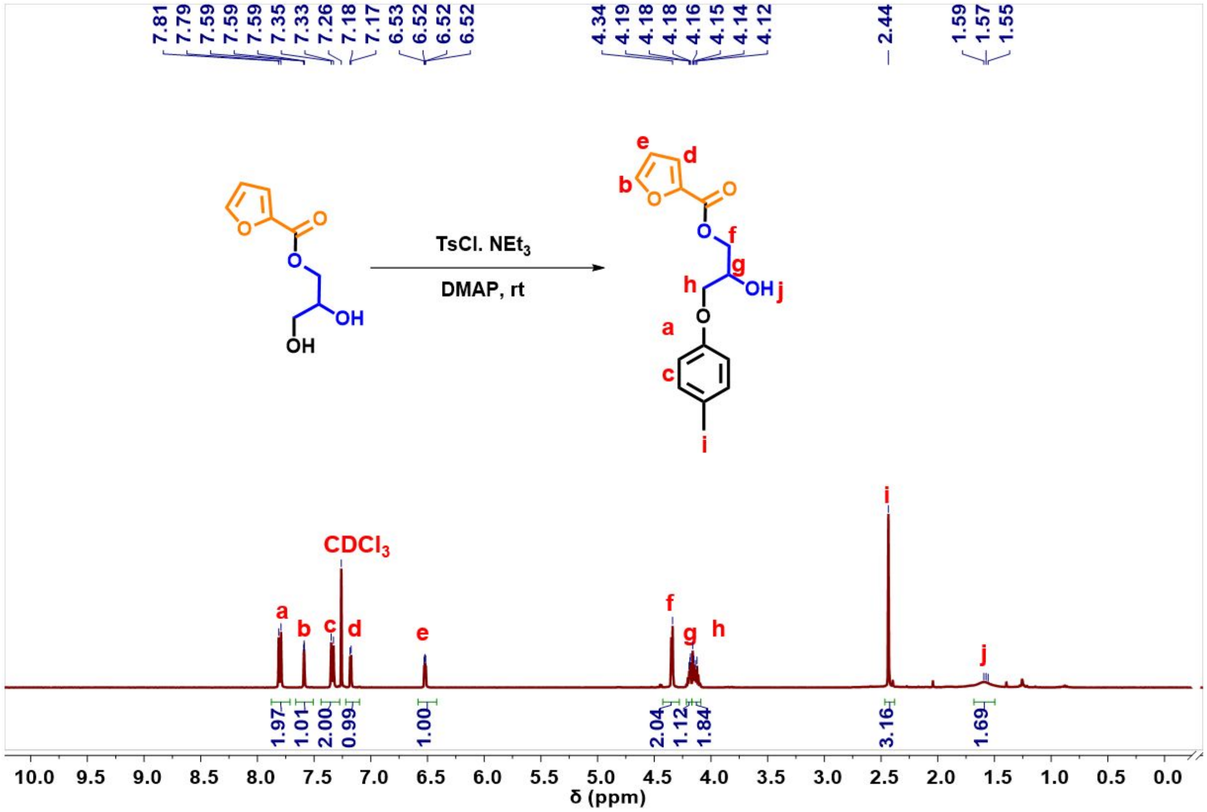

Figure S40 ^{1}H NMR spectrum (400 MHz, $CDCl_3$) of tosylated product obtained from diol.
